# Exome and Transcriptome Sequencing of *Aedes aegypti* Identifies a Locus That Confers Resistance to *Brugia malayi* and Alters the Immune Response

**DOI:** 10.1371/journal.ppat.1004765

**Published:** 2015-03-27

**Authors:** Punita Juneja, Cristina V. Ariani, Yung Shwen Ho, Jewelna Akorli, William J. Palmer, Arnab Pain, Francis M. Jiggins

**Affiliations:** 1 Department of Genetics, University of Cambridge, Cambridge, United Kingdom; 2 Biological and Environmental Sciences and Engineering (BESE) Division, King Abdullah University of Science & Technology, Thuwal, Kingdom of Saudi Arabia; 3 Department of Parasitology, Noguchi Memorial Institute for Medical Research, Accra, Ghana; University of Notre Dame, UNITED STATES

## Abstract

Many mosquito species are naturally polymorphic for their abilities to transmit parasites, a feature which is of great interest for controlling vector-borne disease. *Aedes aegypti*, the primary vector of dengue and yellow fever and a laboratory model for studying lymphatic filariasis, is genetically variable for its capacity to harbor the filarial nematode *Brugia malayi*. The genome of *Ae*. *aegypti* is large and repetitive, making genome resequencing difficult and expensive. We designed exome captures to target protein-coding regions of the genome, and used association mapping in a wild Kenyan population to identify a single, dominant, sex-linked locus underlying resistance. This falls in a region of the genome where a resistance locus was previously mapped in a line established in 1936, suggesting that this polymorphism has been maintained in the wild for the at least 80 years. We then crossed resistant and susceptible mosquitoes to place both alleles of the gene into a common genetic background, and used RNA-seq to measure the effect of this locus on gene expression. We found evidence for Toll, IMD, and JAK-STAT pathway activity in response to early stages of *B*. *malayi* infection when the parasites are beginning to die in the resistant genotype. We also found that resistant mosquitoes express anti-microbial peptides at the time of parasite-killing, and that this expression is suppressed in susceptible mosquitoes. Together, we have found that a single resistance locus leads to a higher immune response in resistant mosquitoes, and we identify genes in this region that may be responsible for this trait.

## Author Summary

Within mosquito populations, genetic differences between individuals affect their ability to transmit human diseases such as malaria, dengue fever, and lymphatic filariasis. In the mosquito *Aedes aegypti*, some individuals are genetically resistant to *Brugia malayi*, a mosquito-vectored parasite that causes a debilitating tropical disease called lymphatic filariasis. To characterize the genetic basis of resistance, we identified resistant and susceptible mosquitoes from a wild Kenyan population, and sequenced the protein-coding region of their genomes (the exome). This allowed us to locate a single region of the mosquito genome that is causing resistance and to identify genes that may be controlling the trait. To understand the mechanisms of resistance, we measured gene expression. The susceptible mosquitoes have reduced expression of immunity genes after they are infected with *B*. *malayi*, including genes known to kill this group of parasites. This is possibly because their immune response is being suppressed by the parasites. We conclude that resistance is controlled by a single locus and show that resistance results in an increased immune response.

## Introduction

The rate at which parasites are transmitted by mosquitoes is an important determinant of the prevalence of vector-borne diseases in human populations. Alongside factors like the number of mosquitoes and their biting preferences, the rate of transmission depends on the ability of mosquitoes to acquire the parasite when feeding on an infected person and subsequently transmit it. This is referred to as their vector competence, and is affected by both environmental and genetic factors [1,2]. Even within a population of a single mosquito species there can be tremendous genetic variation in vector competence, often as a result of differences in the immune response of the mosquito to the parasites they are vectoring [2]. For example, variation exists in susceptibility of *Anopheles gambiae* to the malaria parasite *Plasmodium falciparum* [3,4] and in *Aedes aegypti* to dengue and filarial nematodes [5,6]. This has attracted much attention as it could one day lead to be better disease control by manipulating mosquito populations to reduce vector competence. For example, field trials are underway that are releasing *Ae*. *aegypti* mosquitoes carrying the bacterial symbiont *Wolbachia*, which reduces the mosquitoes’ ability to transmit dengue virus [7].

The tropical disease lymphatic filariasis, or elephantiasis, is a leading cause of morbidity and disability worldwide, with especially high parasite burdens in Africa and south and south-east Asia [8]. It is estimated to affect 120 million people worldwide, and symptoms include lymphedema and swelling of the extremities [9]. In humans, the disease is caused by the filarial nematodes *Wuchereria bancrofti*, *Brugia malayi* and *Brugia timori*, and is vectored by a range of mosquitoes, including species of *Culex*, *Mansonia*, *Anopheles* and *Aedes* [9]. *W*. *bancrofti* is the major cause of filariasis worldwide, leading to 90% of the cases of lymphatic filariasis, and *Brugia* species, which are only found in Asia, cause the remaining 10% [8]. *B*. *malayi* is the main laboratory model for studying lymphatic filariasis, and it grows readily in some strains of the mosquito *Ae*. *aegypti*. Despite having overlapping ranges, *Ae*. *aegypti* does not naturally vector any of the nematodes that cause lymphatic filariasis in humans. It is however a natural vector of *Dirofilaria*, which causes filariasis in dogs [10].

*B*. *malayi*, along with other filarial nematodes, are heteroxenous, requiring both a vertebrate host and a mosquito vector for their life cycle [8,9]. Humans, cats, and monkeys can all serve as vertebrate hosts for *B*. *malayi* [10]. Male and female worms reproduce sexually in the vertebrate, producing microfilariae which circulate in the bloodstream and are ingested by mosquitoes during blood feeding. After penetrating the mosquito midgut, the filarial nematodes develop inside various tissues within the mosquito. In the case of *B*. *malayi*, the microfilariae migrate to the thoracic muscles of the mosquito, where they undergo successive molts until they become L3 larvae [11]. They then migrate to the mosquito proboscis, where they are transferred to the vertebrate host during blood feeding.

Beginning in the 1960’s, mosquito strains and species have been identified that are naturally refractory (resistant) to infection by filarial nematodes [12]. Proposed mechanisms of resistance include reduced ingestion of parasites, physical killing of parasites in the foregut, barriers to penetration of the midgut, and hemolymph factors that kill the parasite in the thoracic cavity and lead to melanotic encapsulation [13]. Some species such as *Armigeres subalbatus*, a natural vector of *Brugia pahangi*, are completely refractory to infection by *B*. *malayi* while being highly susceptible to *B*. *pahangi* [14]. Others, such as *Ae*. *aegypti*, are polymorphic within species for resistance [15]. In laboratory lines of *Ae*. *aegypti*, genetic variation in resistance to *B*. *malayi* has a simple genetic basis, and is primarily determined by a single dominant locus on the first chromosome [16]. This genetic resistance extends to some other species of nematodes, such as *B*. *pahangi* and *W*. *bancrofti*, but not to *Dirofilaria*, for which *Ae*. *aegypti* is a natural vector [16]. In this mosquito, sex is also determined by a region on the first chromosome, and the resistance locus is tightly linked to the sex-determining region [17–19].

The immune responses of *Ae*. *aegypti* have been extensively studied, but it remains unknown which factors are important in killing filarial nematodes and whether genetic differences in susceptibility are caused by differences in immune responses. Despite the mechanisms being unclear, the mosquito immune response does appear to control filarial nematodes. Fewer parasites reached the L3 stage when the immune system was upregulated by inoculating mosquitoes with bacteria before they fed on blood carrying microfilariae [20]. Similarly, parasite numbers were reduced when the mosquito was infected with the bacterium *Wolbachia*, which also upregulated the immune response [21]. Anti-microbial peptide (AMP) production may be responsible for these effects as cecropin negatively affects worm motility [22]. However, activation of the two main immune signaling pathways, Toll and IMD, by RNAi depletion of their negative regulators, *Cactus* and *Caspar*, produced no measurable effect on resistance to *B*. *malayi* [11].

Genetic mapping of parasite resistance in mosquitoes has so far been done by individually testing markers [3,6,18] or with high-density genotyping using SNP arrays or RAD-sequencing [19,23]. These approaches often utilize randomly selected markers sparsely interspersed in the genome and rely on markers being in linkage disequilibrium with the causative polymorphism, which itself is unlikely to be sampled. In species like *An*. *gambiae*, linkage disequilibrium extends very short distances in wild populations [24], and it is preferable to concentrate efforts on regions that are likely to be involved in the trait of interest. In humans, the solution has been to use exome capture to sequence only protein-coding regions of the genome, which has been met with much success in identifying the mutations that cause Mendelian diseases [25]. This is especially desirable in species like humans and *Ae*. *aegypti*, where the large and repetitive genomes mean that whole genome sequencing is prohibitively costly and that much of the non-coding sequence cannot be investigated because relatively short sequence reads cannot be uniquely mapped to the genome.

We have investigated the genetic and mechanistic basis of resistance to *B*. *malayi* in *Ae*. *aegypti* using a combination of genomic and transcriptomic approaches. First, we resequenced the exome using probes we designed for *Ae*. *aegypti* and performed an association study to map the locus causing resistance with unprecedented precision. Using RNA-seq, we then measured gene expression in resistant and susceptible genotypes of the mosquito to understand how this locus alters the transcriptional response to filarial nematode infection. To minimize the contribution of random genetic differences between the resistant and susceptible lines, we performed genetic crosses to isolate the resistance locus in a common genetic background. This allowed us to identify differences in immune and non-immune response gene expression that will facilitate our understanding of mechanisms of resistance.

## Materials and Methods

### Mosquito Strains and Rearing

A wild outcrossed population was established for association mapping. Mosquito eggs were collected in July 2010 from a 120 km stretch between Kilifi, Malindi, and Mombasa in coastal Kenya using oviposition traps [26]. Each trap consisted of a black plastic cup, hay infused water (4 g dried grass in 1 L of water for 4 days) and a strip of creped cardboard paper. Eggs from each collection site (median of 42 eggs/trap with 1–16 traps used per collection site) were hatched in the laboratory and reared separately. Strains were established from two collection sites near Kilifi (St. Thomas and Mabarikani) and one site each near Malindi (Muthangani) and Mombasa (Mtwapa). At the F_2_ generation all strains were reciprocally crossed to each other and to themselves, with similar numbers of males and females in each group. Fifteen males and fifteen females from each cross (480 individuals total) were used to start an outcrossed population, where they were allowed to mate randomly for six generations. Each generation was maintained at a minimum population size of 900 adults and was not allowed to overlap with the previous generation.

We measured the effect of the resistance locus on gene expression by taking advantage of sex linkage to generate susceptible and resistant mosquitoes that are genetically equivalent across most of their genome. Resistance has previously been mapped to approximately 4-21cM from the sex-determination locus [17,19] and is dominant in action. The Liverpool IB12 (LVP-IB12^R^) strain of *Ae*. *aegypti* is a highly inbred line that was used for the genome sequencing project [27] and was previously found to be resistant to infection[19]. It is derived from the Liverpool strain which has been maintained in culture since 1936 and was originally collected from West Africa [12]. A strain of Liverpool susceptible to infection by *B*. *malayi* (LVP-FR3^S^) [19] was obtained from the NIAID/NIH Filariasis Research Reagent Resource Center (FR3, Atlanta, Georgia, USA). We refer to the strains as LVP^R^ or LVP^S^ from this point on. To obtain resistant progeny, we crossed LVP^R^ virgin females to LVP^S^ males and backcrossed F_1_ males to LVP^S^ virgin females. To obtain susceptible progeny, we crossed LVP^S^ virgin females to LVP^R^ males and backcrossed F_1_ males to LVP^S^ virgin females.

All mosquitoes were reared at a larval density of 200 individuals in 1.8 L of water. They were fed liver powder as larvae and 10% w/v fructose with 0.1% para-aminobenzoic acid (PABA) as adults and kept at 28°C (± 1°C) with 75% (±5%) humidity and a 12 hour light:dark cycle. Females were blood fed using an artificial membrane feeder (Hemotek Limited, UK) with donated human blood obtained from Blood Transfusion Services at Addenbrooke’s Hospital, Cambridge, UK. The temperature of the blood was maintained at 37°C in the feeders.

### Infections and Phenotyping

To infect mosquitoes for association mapping, *B*. *malayi* was obtained from Darren Cook and Mark Taylor at the Liverpool School of Tropical Medicine (LSTM), where they were reared in gerbils. Microfilariae were harvested into RPMI medium, which was then centrifuged at 700 rpm for 5 minutes and 0.5 mL of the pellet was transferred to 40 mL of blood. Microfilariae were incubated in the blood at 37°C for at least one hour prior to feeding. Outcrossed and control LVP^S^ mosquitoes were fed on blood containing parasites at a concentration of 457 microfilariae per 20 μl of blood. Female mosquitoes were 6 to 9 days old on the day of infection. Unfed mosquitoes were discarded, and infected mosquitoes were maintained on a 10% fructose solution with 0.1% PABA for 10–11 days post-infection. To check for infection, individual mosquitoes were separated at the head and thorax at 10 or 11 days after infection and incubated in 100 μl of 1X phosphate buffered saline (PBS) for one hour at 37°C. We found this caused L3 larvae to migrate into the PBS and gave similar estimates of infection as individually dissecting mosquitoes. The supernatant was transferred to a microscope slide, the number of L3 parasites was counted, and the mosquito carcasses were stored at -80°C until DNA extraction could be performed. Mosquitoes were classified as susceptible to infection if they had one or more L3 parasites and were classified as resistant if they had none.

For measuring gene expression, resistant and susceptible progeny from the crosses described in the previous section were collected from the following treatments: immediately prior to blood feeding and 12 and 48 hours post-feeding with either a control blood meal or a blood meal containing microfilariae. Microfilariae were harvested into 50 mL RPMI medium and incubated overnight with 0.5 mL gentamicin (10 mg/ml in water) at 28°C, and 0.5 mL of the pellet formed overnight was transferred to 16 mL of blood. The infective blood meal contained 160 microfilariae per 20 ul of blood. A non-infective control of 50 mL RPMI with 0.5 mL gentamicin was also incubated in the same manner, and 0.5 mL of solution was transferred to 16 mL of blood. Both blood vials were then incubated at 37°C for at least one hour prior to feeding. Female mosquitoes were 4 to 8 days old on the day of blood feeding. Three to four replicate cages were maintained for each treatment and all time points were collected from the same cages. After blood feeding, mosquitoes were maintained in paper cups in groups of 8 individuals and were given 10% fructose with 0.1% PABA after collection of the 12 hour time point. We dissected five individual mosquitoes of each genotype at 24, 48, and 72 hours after infection to follow the progression of *B*. *malayi* development in resistant versus susceptible mosquitoes. Pools of 8 individuals for each treatment were snap frozen at each time point and stored at -80°C prior to RNA extraction.

### Library Preparation and Sequencing

DNA was extracted from single mosquitoes using QiaAmp MicroDNA kit (Qiagen) with the following modifications. Tissues were incubated with RNAse post-homogenization and no carrier RNA was used. DNA was eluted in 50 μl AE buffer and 1 μl of eluate was quantified with a Qubit 2.0 fluorimeter (Invitrogen). Total RNA was extracted using Trizol (Invitrogen) and was treated with Turbo DNAse (Ambion) prior to library preparation. RNA integrity was assessed using a Bioanalyzer (Agilent).

We sequenced the exomes of individual mosquitoes. DNA sequencing libraries were made using TruSeq DNA Sample Preparation kits (Illumina). Genomic DNA (600ng to 1ug of starting material) was sheared to 500bp fragment sizes via sonication, and libraries were prepared following the instructions from the manufacturer. Exome capture was then performed to enrich for coding sequences using custom SeqCap EZ Developer probes (Nimblegen). Overlapping probes covering the protein coding sequence (not including UTRs) in the AaegL1.3 gene annotations [27] were produced by Nimblegen based on exonic coordinates specified by us. In total, 26.7Mb of the genome (2%) was targeted for enrichment. Exome capture coordinates are available at https://www.jiggins.gen.cam.ac.uk/data/Aaegypti_exome.bed. Captures were performed on pools of 24 uniquely barcoded individuals, and the target enriched libraries were sequenced with either 100bp paired-end HiSeq2000 or 150bp paired-end MiSeq (see S1 Table). Library preparation, exome capture, and sequencing were performed by the High-Throughput Genomics Group at the Wellcome Trust Centre for Human Genetics (Oxford, UK).

In addition to the exome sequencing, we also produced low coverage whole genome sequences from some mosquitoes (these were largely different individuals but were from the same experiment, see S1 Table). For production of these libraries, DNA was sheared and PCR adapters were added in a single transposase mediated ligation step using the Nextera Library kit (Illumina). Fifty ng of genomic DNA was used per individual and libraries were prepared following the instructions from the manufacturer. Libraries were pooled in groups of 21–25 uniquely barcoded individuals and sequenced with 100bp paired-end HiSeq2000 by the Biosciences Core Laboratory at King Abdullah University of Science of Technology (KAUST) (Thuwal, Saudi Arabia).

RNA sequencing libraries were made using the TruSeq RNA Sample Preparation kit version 1 (Illumina) starting with 3 ug of total RNA per library. Libraries from different treatments were balanced between lanes (see S2 Table), pooled in groups of 8–10 libraries per lane, and sequenced with four lanes of 100 bp paired-end HiSeq2000 by the Eastern Sequence and Informatics Hub (EASIH) (Addenbrooke’s, Cambridge, UK).

### Association Study

Sequences from DNA sequencing libraries were quality trimmed from the 3’ end using Trimmomatic version 0.30 [28] when average quality scores in sliding windows of 4 base pairs dropped below 20 or when the quality score at the end of the read dropped below 20. Sequences less than 50 base pairs in length and unpaired reads were discarded. Sequences were aligned to the reference genome (AaegL1, Oct 2005) [27] with BWA version 0.6.1-r104 [29] using the default parameters. Alignments for individuals sequenced across different lanes were merged into single BAM files using Picard version 1.93. Alignments were sorted, indexed, and assigned read groups using SAMtools version 0.1.18 [30] and Picard. Indels were realigned using GATK version 2.3 [31], and PCR and optical duplicates were removed using Picard. We have deposited the raw sequencing reads to the Short Read Archive with Accession Number SRP044393.

We performed association mapping using a combination of high and low coverage sequences. Average exome coverage from whole genome sequenced libraries was 0.73X per sample while average exome coverage from exome captured libraries was 32X for HiSeq sequencing and 2.3X for MiSeq sequencing. For this reason, we tested for associations with infection status using genotype posterior probabilities, which incorporate uncertainty in genotype calls, rather than calling individual genotypes prior to mapping [32] using the doAsso function in ANGSD version 0.539 [33]. BAM files were used as input for ANGSD. All SNPs called with a LRT statistic greater than 24 (*P*<10^–6^) were tested for association with susceptibility to *Brugia*. Only bases with a minimum base quality greater than 20 and only reads that were uniquely mapped and with a mapping quality greater than 20 were included. Major and minor alleles were inferred from genotype likelihoods using the genotype likelihood model implemented in SAMtools [34], and allele frequencies were estimated assuming a known minor allele using an EM algorithm [35].

Associations were tested under an additive model with logistic regression, a dominance model or a recessive model. The dominance and recessive models test for associations with infection status assuming the minor allele is dominant or recessive respectively. In addition, the additive model was reimplemented setting the most significant marker from the original test as a covariate (supercont1.398, position 175496) to test for the presence of a second locus. Only individuals with full genotypic information at this SNP with a posterior probability of 0.7 were included (73 of 140 individuals), and the covariate was coded under the dominant model. At least 15 individuals were required to have each genotypic class for the additive, dominance, and covariate models, and at least 10 individuals were required to have each genotypic class for the recessive model. To obtain a genome-wide significance threshold for each model that is corrected for multiple tests we permuted the phenotypes and repeated the analysis 200 times, each time retaining the lowest *P*-value across all variants to generate a null distribution. This was used to set a genome-wide significance cutoff of *P*<0.01 and *P*<0.05. We also tested whether any indels were associated with resistance. ANGSD can only test SNPs for associations directly from BAM files, so we provided indel genotype probabilities, which are used in an intermediate step in ANGSD, to test for associations. Genotype probabilities for indels were produced using GATK’s UnifiedGenotyper and ProduceBeagleInput. Only the additive model was tested using this method, and significance was assessed by permutation as described for SNPs. The variant effect predictor [36] was used to assign variants to genes and classify their effects (non-synonymous, synonymous, etc) using gene annotation set AaegL1.3.

We found that multiple SNPs in linkage disequilibrium were associated with resistance, so we excluded variants that explained the infection data significantly less well than our top hit (supercont1.398, position 175496). Using only the HiSeq exome sequenced individuals, we fitted a generalized linear model with a logit link function, where the response was the probability of a mosquito being infected with an L3 worm, and the predictor variables were the ‘top hit’ SNP and the SNP in question. A SNP was rejected if it was not significant but the ‘top hit’ SNP was.

### RNA-seq Analysis

Sequences from RNA sequencing libraries were quality trimmed using the same method as used for DNA sequencing libraries, except that sequences less than 25 base pairs in length were discarded. An average of 42 million paired-end reads were obtained from each of the 36 libraries (S2 Table). Reads were aligned to predicted transcripts in the *Ae*. *aegypti* transcriptome (gene annotation set AaegL2.0) with Bowtie2 version 2.1.0 [37] using TopHat2 version 2.0.9 [38] with 10 mismatches allowed, read gap length and read edit distance set to 5, and no novel junctions allowed. Reads were mapped to the *B*. *malayi* genome (Ensembl version 3.0.19) [39] using TopHat2 as described above, but gene expression was not analyzed further due to low coverage. We have deposited the raw sequencing reads to the Short Read Archive with Accession Number SRP044393.

Differential expression analysis was performed using edgeR [40] after enumerating the number of reads per transcript with HTSeq [41]. We made the following comparisons: 1) Differential expression in response to infection, performed separately for each genotype and time point; 2) Differential expression between genotypes prior to infection to measure constitutive expression differences; 3) Difference in response to infection between genotypes (interaction model), performed separately for each time point. In all cases, we filtered out lowly expressed genes by requiring that each gene included in our comparison have at least 0.1 count per million (0.1 cpm) in enough samples to equal our smallest replicate size for that comparison (n = 2–4). All pairwise comparisons were made using exact tests, and the interaction models were fit using general linear models that accounted for genotype and infection status. Significance was assessed either as having an experiment-wide FDR<0.20 (pairwise comparisons) or an individual gene significance of *P*<0.01 (interaction model). The biological coefficient of variation (BCV), a measure of biological variability between replicates, ranged from 0.179 to 0.455 (S3 Table). After excluding the four libraries with the lowest library amplifications (S2 Table), the BCV ranged from 0.179 to 0.353. The number of genes meeting our filtering criteria ranged from 12,549 to 13,594 (of 17,165) after excluding poor libraries (S3 Table).

We used the RNA-seq data and Popoolation2 [42] to measure differentiation (*F*_ST_) between resistant and susceptible progeny on a per SNP and per gene basis. BAM alignments from all treatments from the same cross (yielding either resistant or susceptible progeny) were merged prior to analysis.

We compared our data on gene expression patterns with previously published microarray data for the Toll and IMD pathways [43] and JAK-STAT pathway [44]. To determine which pathways were activated by infection, we examined gene expression patterns in response to *B*. *malayi* in those genes that were previously shown to be differentially expressed as a result of perturbation of each pathway. We also compared expression patterns with the response to infection by *Wolbachia* strain wMelPop-CLA [45]. Data for this comparison was downloaded from VectorBase [46], and only genes that were significant at *P*<0.01 were used for comparison. We classified immunity genes using ImmunoDB (http://cegg.unige.ch/Insecta/immunodb/) and manual curation by ourselves of more recently identified immune genes.

## Results

### Exome Sequencing of a Kenyan *Ae*. *aegypti* Population Identifies a Single Dominant Locus That Controls Susceptibility to *B*. *malayi*

To create a population that varied in susceptibility to *B*. *malayi*, we collected *Ae*. *aegypti* eggs from the coastal region of Kenya where there is known to be a mixture of genetically resistant and susceptible individuals [15]. These eggs were used to create a large outcrossed population that was maintained in the laboratory for 6 generations. The mosquitoes were then fed on human blood containing *B*. *malayi* microfilariae, which resulted in 23% (88 of 388) becoming infected with L3 larvae, with an average of 2.4 L3’s in each infected mosquito. This is a considerably lower infection rate than in the susceptible control line (86% of mosquitoes infected, 19 of 22, with an average of 2.5 L3’s per infected mosquito), suggesting that there is genetic variation in susceptibility within our population.

To identify genes associated with resistance, we used a combination of exome sequencing or low coverage whole-genome sequencing. The *Ae*. *aegypti* genome is large and repetitive, so exome sequencing provided us with far higher coverage of the exonic regions than was possible with the whole genome sequencing. The exome capture was highly efficient, resulting in 100 times greater coverage of the exome regions (26.7 MB, 2% of the genome) compared with the non-exome regions. So that we could combine the high and low coverage data, we performed our association mapping using an approach based on genotype probabilities at each site (as opposed to calling genotypes and then testing each site for an association with infection). In total we sequenced 67 L3-infected and 73 uninfected mosquitoes, which we classified as susceptible and resistant respectively.

**Fig 1:**
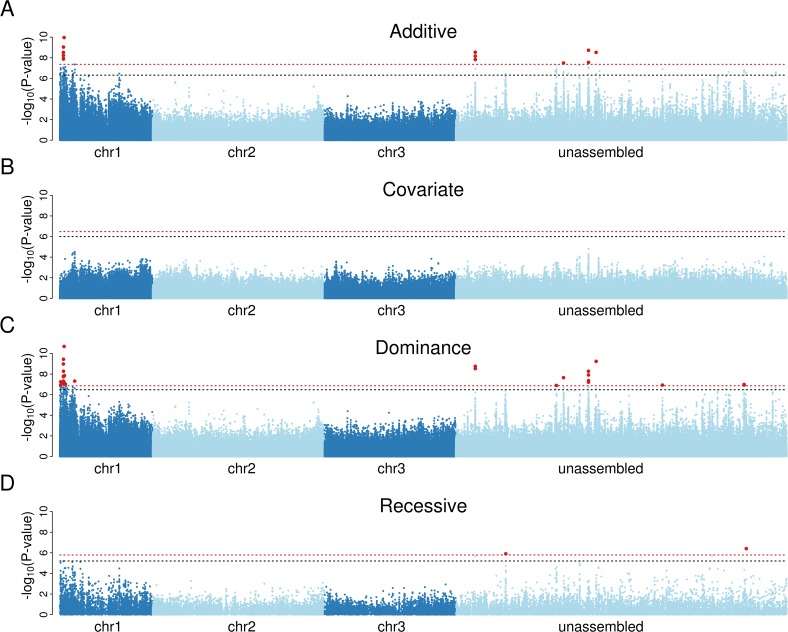
Association mapping demonstrates that a single dominant locus controls resistance to *B*. *malayi* in a Kenyan population of *Ae*. *aegypti*. A) Mapping with an additive model of inheritance produces a small number of significant associations on chromosome 1 and in regions unassigned to chromosomes. B) Including the most significant marker in panel A as a covariate in the analysis results in no significant associations, suggesting that a single locus controls infection. C) A dominance model of inheritance produces highly significant associations, whereas D) a recessive model does not. Red-dotted line shows genome-wide significance at *P*<0.01; black-dotted line shows significance at *P*<0.05. The x-axis represents a physical map (bp) made by arranging scaffolds along the genetic map [19], with scaffolds mapping to the same genetic map position being ordered randomly. To correct genome misassemblies, scaffolds that contain any gene with at least 10 segregating sites and an *F*_ST_ greater than 0.1 in the RNA-seq data were moved to the unassembled region. In the dominance model, 13 sites on 4 scaffolds map to 0 cM and a single site maps to 12 cM.

We found that susceptibility to *Brugia* has a simple genetic basis in our population, with a small number of sites highly significantly associated with infection (Fig. 1A). Of the sites that have a known position in the genome, all of those with a genome-wide significance of *P*<0.01 were clustered together at 0 cM on chromosome 1 (Fig. 1A). To test whether there were multiple genes affecting susceptibility to *Brugia*, we repeated the association study including the most significant variant from the first analysis as a covariate. This resulted in no significant associations (Fig. 1B). Furthermore, quantile-quantile (qq) plots comparing expected and observed *P*-values in the analysis with the top SNP as a covariate confirm that there are no additional associations (S1 Fig). Therefore we can conclude that there is a single variant causing the differences in susceptibility, and all the significant associations are caused by sites in linkage disequilibrium with this variant.

To test whether resistance is dominant, we repeated the association study using a model that assumed the minor allele to be either dominant or recessive. The dominant model resulted in a cluster of significant associations on chromosome 1, and the top associations were more significant than the previous analysis that assumed additive effects (Fig. 1C). In contrast, the recessive model generated only two marginally significant associations (Fig. 1D), and inspection of these showed that they were caused by linkage disequilibrium with the highly significant dominant variants. The minor allele of the most significant site was associated with increased resistance, so we can conclude that resistance is caused by a single dominant locus at 0 cM on chromosome 1 (positions 1p23 and 1p25 on the physical map [47]). This is within the same region that we previously genetically mapped in crosses between laboratory lines from West Africa (0 to 12 cM) [19], and in the same physical region where a sex-linked, dominant resistance locus was approximately mapped (resistance: 1p31, sex determining region: 1q21) [18,48], so the associations we detected are likely to be caused by the same resistance region. Therefore, the previously identified laboratory QTL is present at an appreciable frequency in East Africa in the wild.

### Identification of Candidate Genes Associated with Resistance

We next examined whether the variant causing resistance could be identified in our dataset. We found 26 SNPs associated with infection below a genome-wide significance threshold of 1% and an additional 27 below 5% (Dominance Model; Fig. 1C and S1 Dataset). No indels were significantly associated with infection. Because of our much higher coverage of the exome, all of the significant associations were in or near to genes.

We used two criteria to exclude SNPs from the list of candidate loci. First, our data only provides evidence for a single causative variant, so we are able to reduce this list from 53 SNPs to 19 SNPs by excluding variants that explain the phenotypic data significantly less well than our top hit (see methods for details; based on HiSeq exome sequences alone, S1 Dataset). Second, in the experiments described below we find that worms never develop in mosquitoes carrying the resistance allele, and only 7 of the 19 SNPs follow this pattern (allowing a 10% phenotyping error and using only HiSeq exome data, S1 Dataset). None of these SNPs alter the protein sequence (6 synonymous and 1 intronic, S1 Dataset). Furthermore, these SNPs occur in five different genes, none of which have known immune functions (gamma-tubulin complex component 3 [AAEL008465], cysteine synthase [AAEL008467], putative latent nuclear antigen [AAEL002082], conserved hypothetical protein [AAEL008350], and chaoptin [AAEL008940]). Therefore, we cannot identify a single variant that causes resistance.

A combination of two factors has likely prevented us from identifying the gene that is causing mosquitoes to be resistant. First, linkage disequilibrium in our mapping population means that significant associations are found across multiple scaffolds, sometimes with identical segregation patterns. This is visible in quantile-quantile plots where there is a large excess of sites with differing frequencies in resistant and susceptible chromosomes (S1 Fig). Second, low sequencing coverage means we have limited power to detect associations in non-coding sequence. Therefore, if the variant causing resistance is in a low coverage region we may not detect it, but it will generate significant associations in nearby genes in linkage disequilibrium.

While we did not identify a causal variant, the list of sites and genes associated with resistance are strong candidates. Even if none of these genes prove to be causing resistance, we have narrowed the region down to the 14 scaffolds that either map to the 0 cM genetic position on chromosome 1 (scaffolds 1.48b, 1.97b, 1.166, 1.222a, 1.267, 1.272, 1.296, 1.327, 1.398, 1.461, 1.487, 1.512, 1.696, and 1.970, containing 175 genes and 15.0 Mb) or the 6 scaffolds with SNPs in the top 1% of hits whose location in the genome is uncertain (scaffolds 1.226, 1.360, 1.676, 1.1219, 1.257, 1.389, containing at most 94 genes and 6.6 Mb). *F*_ST_ data obtained from our RNA-seq experiment (described in the following section) supports four of these six scaffolds as being located on chromosome 1 (1.360, 1.676, 1.257, and 1.389). This conflicts with a previous analysis showing that two of these scaffolds (1.360 and 1.257) are on chromosome 2 [19], but this is not unexpected as the genome has a high misassembly rate, and many scaffolds are chimeras of sequences found in different places in the genome [19].

### *Brugia malayi* Development in Resistant and Susceptible Genotypes of *Ae*. *aegypti*

To examine the effects of the *B*. *malayi* resistance locus on mosquito gene expression and worm development we used two laboratory mosquito lines, one resistant (LVP^R^) and one susceptible (LVP^S^). When comparing these lines it is desirable to homogenize the genetic background in which the two alleles of the locus are found. To do this we took advantage of the locus being dominant and sex-linked. By crossing resistant (LVP^R^) males with susceptible (LVP^S^) females or vice-versa, and then backcrossing the F_1_ males to susceptible female (LVP^S^) mosquitoes, we generated resistant and susceptible mosquitoes that are genetically similar except for the effects of other dominant sex-linked genes. We checked that this was the case by calling SNPs from the RNA-seq data (described below) and measuring differentiation between the backcross progeny using *F*_ST_, which ranges from 0 (complete allele sharing) to 1 (complete differentiation). As expected, we found that the first chromosome was differentiated between the backcross progeny from the resistant grandmother compared to progeny from the susceptible grandmother (mean *F*_ST_ = 0.081). The second and third chromosomes were not differentiated (mean *F*_ST_ = 0.011 and 0.009 respectively) (S2 Fig). The region of differentiation extended across the length of the first chromosome (S2 Fig), which is likely to be due to the low rate of recombination in *Ae*. *aegypti*.

**Fig 2:**
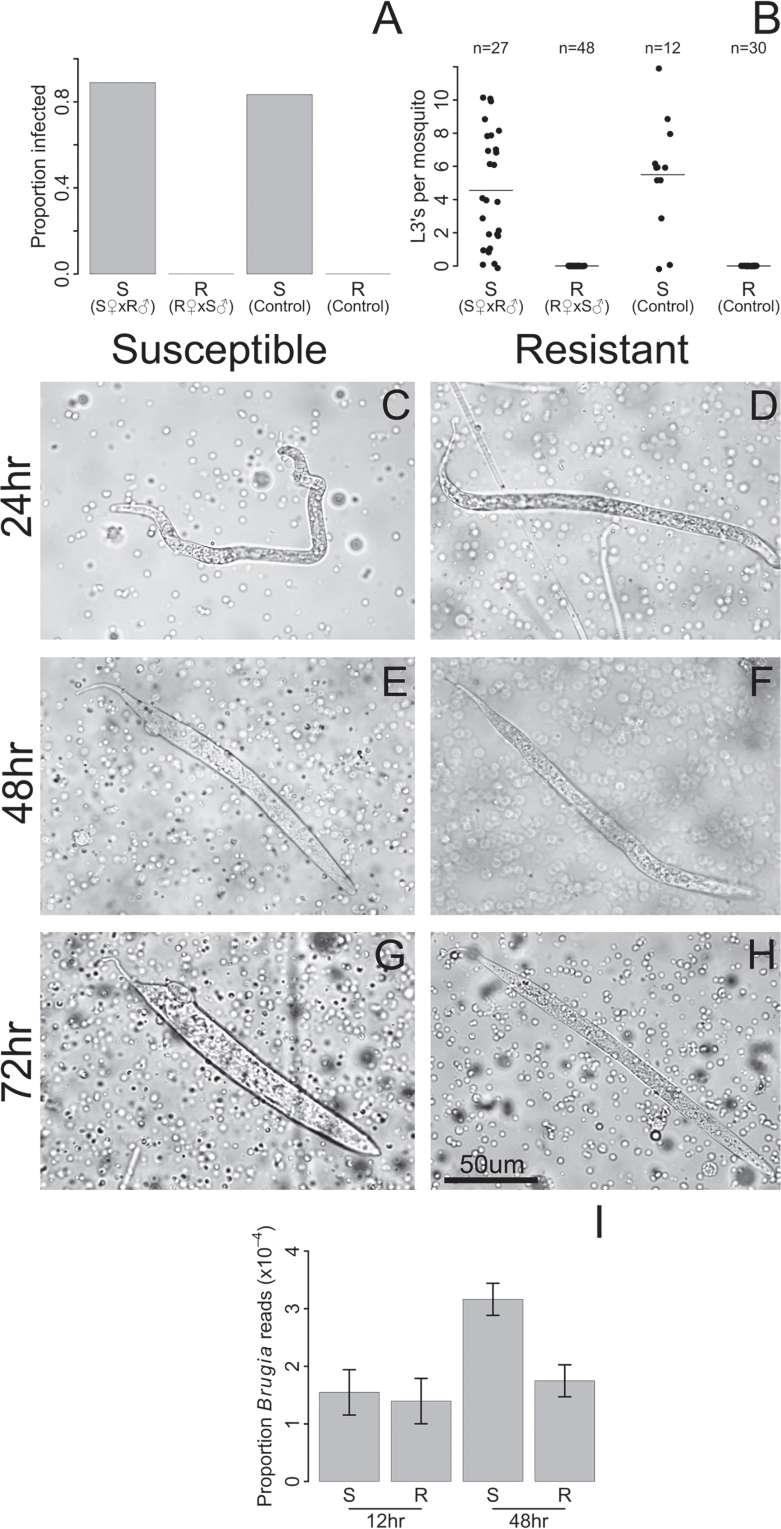
Resistance to *B*. *malayi* is dominant and sex-linked, and it impairs early stages of *B*. *malayi* development. Female progeny from a backcross inherited the resistance phenotype of their maternal grandparent, whether resistance was measured as A) the proportion of mosquitoes infected with L3 stage parasites or B) the number of L3 stage parasites at 10 days after infection. S♀ x R♂ (referred to as susceptible from this point forward) were created by crossing an LVP^S^ female with an LVP^R^ male, followed by backcrossing the F_1_ progeny to LVP^S^. R♀ x S♂ (referred to as resistant) were created in the same way except an LVP^R^ female was crossed with an LVP^S^ male in the parental generation. By 24 hours after infection, many *B*. *malayi* microfilariae have migrated from the midgut to the thoracic tissues in both C) susceptible and D) resistant hosts. At 48 hours after infection, microfilariae are molting into the non-feeding L1 developmental stage in E) susceptible hosts, whereas growth is arrested in F) resistant hosts. By 72 hours after infection, nearly all surviving *B*. *malayi* are in the L1 stage in G) susceptible hosts, whereas they are dead or dying in H) resistant hosts. I) Genome-wide gene expression levels of *B*. *malayi* are comparable in resistant and susceptible hosts at 12 hours after infection, whereas by 48 hours, gene expression, and presumably growth, of *B*. *malayi* is higher in susceptible mosquitoes. *B*. *malayi* gene expression is estimated by dividing the total number of RNA-seq reads mapping to the *B*. *malayi* genome by the total number of reads mapping to the *Ae*. *aegypti* genome.

We found that resistance closely segregated with the sex-determining locus (Fig. 2 A-B), with no L3 worms (0 of 48) developing in the backcross progeny from the resistant (LVP^R^) grandmother. In contrast, in the backcross progeny from the susceptible (LVP^S^) grandmother, 89% (24 of 27) of the mosquitoes were infected, and these had an average load of 5.1 L3’s per mosquito (Fig. 2 A-B). The infection rates of these backcrosses were similar to those of the parental lines (LVP^S^ and LVP^R^; Fig. 2 A-B). The sex determining locus is at approximately 21 cM [19], and we observe no recombinants between the sex and resistance loci (0 of 48, Fig. 2 A-B), although some recombinants may not be detected because not all susceptible individuals develop an infection (83%, 10 of 12). These results suggest that the difference in resistance between these lines is being controlled by the same dominant sex-linked locus described in the Kenyan population in the previous section. We will subsequently refer to these as the resistant and susceptible genotypes.

Using these genetically similar mosquitoes we examined the stage in *B*. *malayi* development being targeted by resistant mosquitoes. In susceptible *Ae*. *aegypti*, microfilariae penetrate the midgut and migrate to the thorax from 1–24 hours following infection, enter a non-feeding L1 stage of development from 6–72 hours, and eventually exit the mosquito as L3 larvae after approximately ten to twelve days [11,49]. Microfilariae are approximately 200–300 um in size at the time of ingestion and L3’s can reach 1–3 mm in size [11], and the numbers of worms decrease from an average of 17 microfilariae per mosquito at the time of ingestion to 6 at the L3 stage [11]. We find microfilariae and L1’s in the thorax of both resistant and susceptible mosquitoes for several days after infection (Fig. 2 C-H). At 24 hours we found that microfilariae have migrated from the midgut to the thoracic tissues in both genotypes (Fig. 2 C-D). We continue to find live microfilariae and L1’s in both genotypes at 48 and 96 hours (Fig. 2 E-H), which is consistent ongoing migration until blood digestion ends at around 72 hours after feeding [50]. By 48 hours and especially by 96 hours after infection there are clear differences between the genotypes, with microfilariae molting into the non-feeding L1 larvae in susceptible hosts, whereas growth is arrested in resistant hosts (Fig. 2 E-H). The microfilariae and L1’s in the resistant genotype are phenotypically distinguishable from those in susceptible genotype, and appear to be smaller and thinner (Fig. 2 G-H). We could not accurately compare microfilariae numbers using our dissections, so we instead compared gene expression (see next paragraph). We sometimes see worms being melanized in both genotypes, but usually they are not.

We can also follow worm development from the number of RNA-seq reads mapping to the *B*. *malayi* genome. At 12 hours, *B*. *malayi* gene expression levels are comparable in resistant and susceptible genotypes, suggesting similar growth and numbers at this time point. By 48 hours, gene expression is much higher in the susceptible genotypes, suggesting greater parasite growth in the susceptible host [51] (Fig. 2I). This suggests that parasite development is aborted in resistant mosquitoes between 12 and 48 hours after infection, although languishing microfilariae and L1’s can be found in resistant mosquitoes for several more days. Our results corroborate previous studies which show that parasites in refractory *Ae*. *aegypti* decline in numbers by 48 hours after infection [49,51,52]. Therefore, parasites are able to reach thorax of resistant mosquitoes, but are killed in the microfilariae and L1 stages. This suggests that the resistance mechanism plays an important role after invasion of the thorax.

### Resistant and Susceptible Mosquitoes Have a Similar Transcriptional Response to Infection by *B*. *malayi*

**Fig 3:**
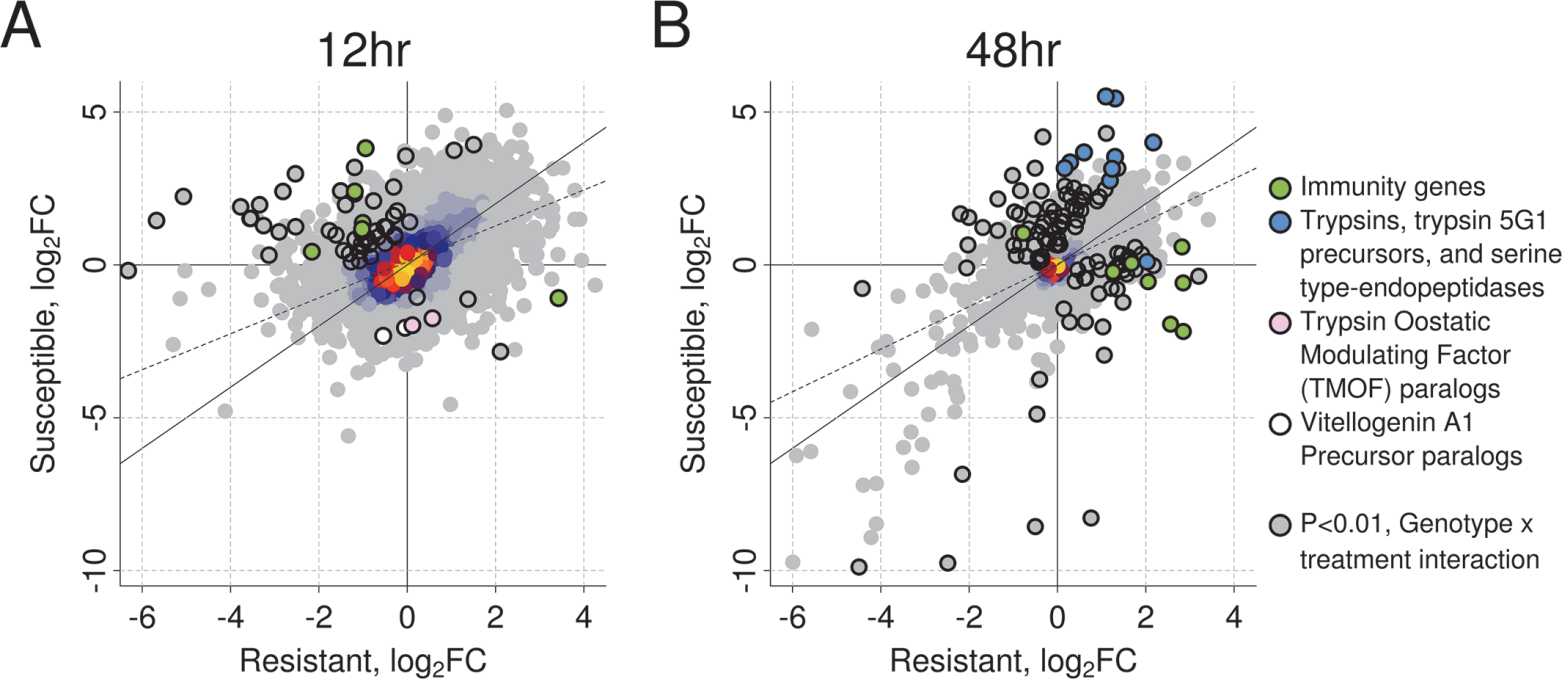
The induced response to *B*. *malayi* is highly correlated between resistant and susceptible mosquitoes. The correlation in log_2_ fold change between infected versus uninfected mosquitoes is shown at (A) 12 hours (Pearson's R^2^ = 0.51) and (B) 48 hours (Pearson's R^2^ = 0.58) post infection for resistant and susceptible mosquitoes. The genes highlighted with black circles differ in the direction or magnitude of expression (interaction term significant at a *P*<0.01). The black diagonal line shows the relationship expected with a perfect correlation, and the dotted line shows the best fit line from the observed data. Warmer colors (uncircled) indicate a higher density of superimposed points.

We used RNA-seq to investigate the transcriptional response of *Ae*. *aegypti* to *B*. *malayi* (using mosquitoes from the same experiment as shown in Fig. 2). We chose to measure gene expression early in the infection when differences in worm development in susceptible and resistant mosquitoes appear. For each genotype separately, we measured differential expression between infected and uninfected mosquitoes. We found a strong correlation between genotypes in the induced responses at both time points (Pearson’s *R* ^*2*^ at 12hr: 0.51, 48hr: 0.58; Fig. 3), suggesting a similar response to *B*. *malayi* infection in resistant and susceptible mosquitoes.

Our first time-point was 12 hours post-infection, when microfilariae are migrating from the midgut to the thorax and beginning to enter the L1 stage. *B*. *malayi* infection significantly regulated 459 genes in the resistant line (238 induced and 221 repressed) and 636 genes in the susceptible line (535 induced and 101 repressed) (S2 Dataset). A total of 97 genes were significantly upregulated in both resistant and susceptible mosquitoes in response to infection, and 26 were significantly downregulated in both genotypes.

Our second time point was 48 hours post-infection, when over half of the parasites will be L1’s in susceptible mosquitoes [11] and parasites are beginning to die in resistant mosquitoes. At this time, *Brugia* infection significantly regulated 1,029 genes (725 genes induced and 304 genes repressed) in the resistant line and 609 genes (467 genes induced and 142 genes repressed) in the susceptible line (S2 Dataset). A total of 274 genes were significantly upregulated in both genotypes and 91 genes were downregulated in both genotypes.

### Similarities in the Immune Response of Resistant and Susceptible Mosquitoes following Infection by *B*. *malayi*

**Fig 4:**
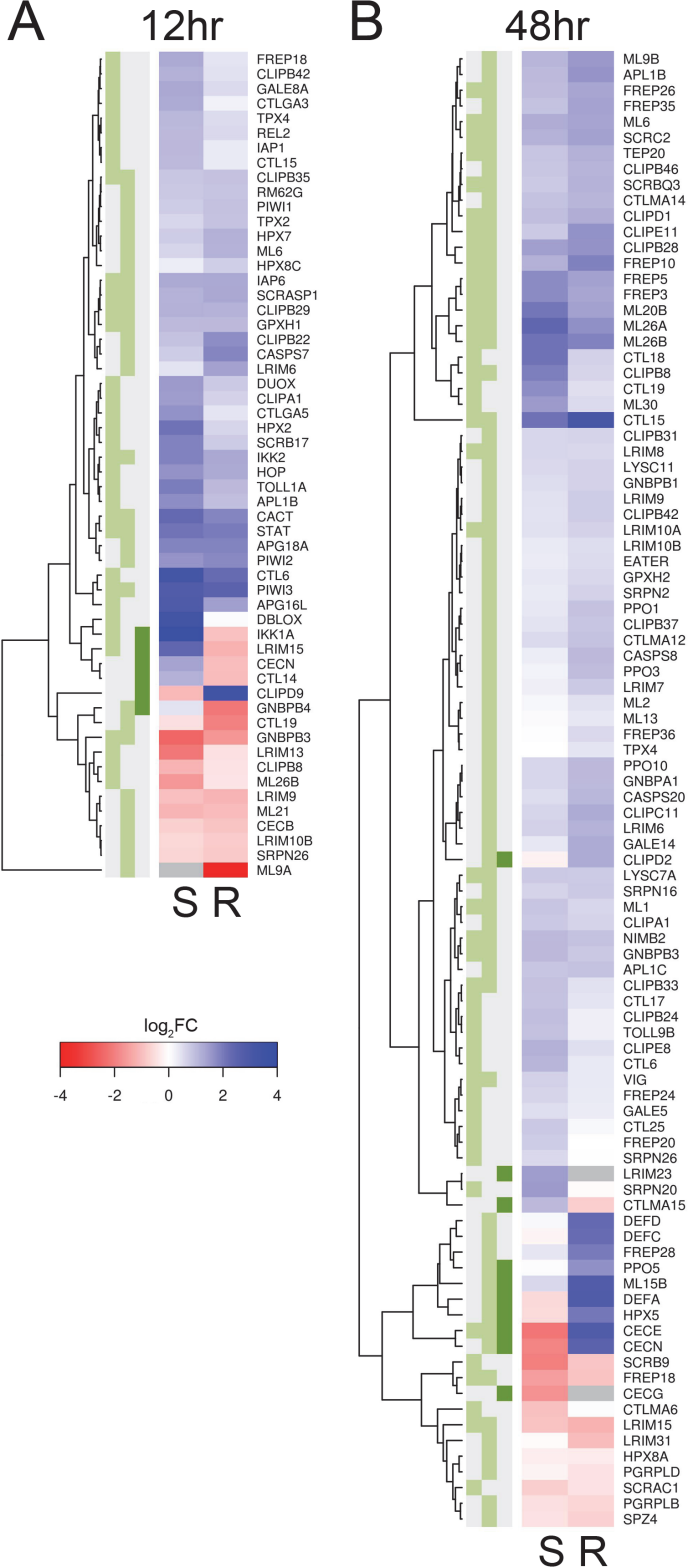
Immune-related genes that are differentially expressed in response to *B*. *malayi* infection. Immune-related genes are from the ImmunoDB database or manual curation, and only those that are significantly differentially expressed for either or both genotypes (or have a significant interaction term) are shown. Expression is log_2_ fold-change in response to *B*. *malayi*, with genes in blue being upregulated and genes in red being downregulated. Grey means insufficient coverage to estimate expression. (A) At 12 hours post infection, almost all genes are being expressed in the same direction and with similar magnitudes. (B) At 48 hours, a subset of genes are being expressed differently in response to infection. The green boxes indicate statistical significance in susceptible mosquitoes (left, FDR<0.2), resistant mosquitoes (middle, FDR<0.2) or a significant interaction (dark green right, *P*<0.01).

There is a clear transcriptional response of immune-related genes to *B*. *malayi* at both 12 and 48 hours after infection (Figs. 3, 4). The large majority of the immune-related genes are upregulated in the infected relative to the uninfected mosquitoes, indicating that the immune system is being activated (Fig. 4). Furthermore, this upregulation of immune genes is apparent at 12 hours post-infection, which conflicts with an earlier report that very little transcriptional response is seen until 5 days after infection in susceptible mosquitoes [11]. The previous study used microarrays and pooled across several time points so may be less sensitive than our approach.

There is a striking concordance between the transcriptional response of immune genes in the resistant and susceptible genotypes (Fig. 4). Among the immune genes significantly differentially regulated 12 hours post-infection in at least one of the genotypes, almost invariably the gene is differentially regulated in the same direction in the other genotype (even if this is not statistically significant) (Fig. 4). At 48 hours there is still a similar response in the two genotypes, although some differences are emerging (see below).

The genes that are differentially regulated in response to *Brugia* infection in both genotypes have a range of immune functions (Fig. 4). These include melanization (*CLIPB8*, *CLIPB29*, and *CLIPB35* [53]), hemocyte-mediated immunity (*CLIPD1*, *FREP3*, *FREP5*, *FREP10*, and a Nimrod homolog [54]), antioxidant protection (*GPXH1*, *HPX3*), the Toll pathway (*CACT*, *GNBPB3*), IMD pathway (*IKK2*), and JAK-STAT pathway (*STAT*). Melanization of *B*. *malayi* was occasionally observed at early stages of infection in both genotypes and is controlled by clip serine protease cascades and phenoloxidases which are released by hemocytes [53]. It is associated with the production of reactive oxygen species, which is expected to trigger the production of antioxidants. The activation of the Toll, IMD and JAK-STAT pathway by *Brugia* infection has never been previously described.

### A Small Number of Genes Respond Differently to Early Stages of Infection in Resistant and Susceptible Mosquitoes

In the resistant and susceptible mosquitoes a subset of genes responded to *B*. *malayi* infection in different directions or with a significantly different magnitude, and these may give clues as to the mechanism of resistance (Figs. 3, S3). At 12 hours post-infection we detected 86 such genes (*N* = 13,156) by testing for a significant interaction between genotype and infection (S2 Dataset). Of these, 62 had sufficient data to be tested for differential expression separately within each genotype. Contrary to our expectation, we found that most (53 of 62) of these genes were more highly expressed in the susceptible line, with 48 of these being upregulated in the susceptible line and downregulated in the resistant (Fig. 3).

There is little evidence to suggest that resistance is due to immune gene expression at the early 12 hour time point, as the susceptible mosquitoes upregulated more immune-related genes than the resistant ones (Figs. 3, 4). Immune genes upregulated in susceptible and downregulated in resistant mosquitoes include a cecropin (*CECN*), a lectin (*CTL14*), a gram-negative binding protein (*GNBP4*), the IMD signaling pathway molecule *IKK1A*, and a leucine-rich repeat protein (*LRIM15*), all of which may be related to the immune response (Fig. 4). In contrast, the resistant line only upregulated expression of a single clip-domain serine protease (*CLIPD9*) (Fig. 4). Furthermore, 10 genes that are induced by infection by the *Wolbachia* strain wMelPop-CLA, which is known to make mosquitoes more resistant to filarial nematodes [21,45], are upregulated in the susceptible line but downregulated in the resistant line (S5 Fig).

At the early time point, the non-immunity genes that had a different transcriptional response to *B*. *malayi* in the two genotypes had a diversity of functions, but many were related to metabolism, with the susceptible line upregulating transport and digestion-related genes and the resistant line downregulating digestion and upregulating vitellogenin (egg yolk protein) (Figs. 3, S4). Of the 48 genes upregulated in the susceptible genotype and downregulated in the resistant genotype, a number were transporter, transmembrane, or gut structural genes (amino acid transporter *PAT1* (AAEL007191), innexin (AAEL014846), integrin (AAEL014846), an insect major allergen/G12 family member (AAEL009166), and a monocarboxylate transporter (AAEL013915)). A translational repressor that is important in starvation responses was also induced only in the susceptible line (eukaryotic initiation factor 4E binding protein, AAEL001864) [55]. Only nine genes had higher induction in the resistant genotype. Two were paralogs of a vitellogenin A1 precursor (AAEL006126 and AAEL006138) and two were paralogs of trypsin modulating oostatic factor (TMOF) (AAEL006670 and AAEL014561). TMOF is expressed in the ovaries and binds to receptors in the midgut where it turns off trypsin production [56] and downregulates the production of vitellogenin. The other genes were an immune modulator (CLIPD9), an AMP dependent ligase (AAEL006823), a putative pupal cuticle protein (AAEL010127) and two genes with unknown functions (AAEL008449 and AAEL000681).

### Susceptible Mosquitoes Downregulate Some Immune-Related Genes 48 Hours after *B*. *malayi* Infection

At 48 hours post-infection, the time point where parasites are being killed in resistant but not susceptible mosquitoes, 152 genes (out of 13,594) responded to *B*. *malayi* infection differently in the susceptible and resistant mosquitoes (Figs. 3, S3 and S2 Dataset). Of the 122 genes with sufficient data to be tested for differential expression in response to infection within genotype, 75 had higher expression in the susceptible line and 47 had higher expression in the resistant line.

In contrast to the earlier time-point, by 48 hours there were differences in the induced immune response between resistant and susceptible mosquitoes. The 152 genes that behaved differently between the two genotypes were significantly enriched for immune response categories (S4 Fig) and these immune response genes tended to have higher expression in the infected resistant mosquitoes (Figs. 3, 4, S3). These included antimicrobial peptides (*CECE*: AAEL000611, *CECN*: AAEL000621, *DEFA*: AAEL003841, *DEFC*: AAEL003832, and *DEFD*: AAEL003857), a prophenoloxidase (*PPO5*: AAEL013492), *JNK* (AAEL008622), *CLIPD2* (AAEL004979), FREP28 (AAEL003156), a Niemann Pick-type C gene *ML15B* (AAEL009556), and the heme peroxidase *HPX5* (AAEL002354). The only immune gene that had higher expression in susceptible mosquitoes was *DSCAM* (AAEL011284). Unexpectedly, many immune genes were downregulated in susceptible mosquitoes in response to *B*. *malayi* infection while being upregulated in the resistant ones (Fig. 4). Two Cecropin genes were found to be suppressed in susceptible mosquitoes at 48–72 hours post-infection in a previous study [11], supporting our results. This pattern therefore suggests parasite suppression of these immune response genes or dysregulation within susceptible mosquitoes.

At 48 hours after infection, the susceptible genotype appears to have higher upregulation of genes that are highly induced by blood feeding [50] and are potentially related to metabolism and digestion. This include trypsins (AAEL013703, AAEL013707, AAEL013712, and AAEL013715) [57], several chymotrypsin-related serine proteases expressed in the midgut (AAEL001674, AAEL001690, AAEL001693, AAEL001701, AAEL008769, and AAEL008780) [57], a putative serine collagenase (AAEL007432) [57], a sphingomyelin phosphodiesterase (AAEL013717) [27], and methylenetetrahydrofolate dehydrogenases (AAEL006085 and AAEL014871) [27]. This is consistent with trypsin modulating oostatic factor (TMOF) paralogs, which inhibit late trypsin production, being downregulated in the susceptible line but not the resistant line at 12 hours, leading to a longer period of expression of trypsins [56].

### The Transcriptional Response to *B*. *malayi* Implicates the Toll, IMD and JAK-STAT Pathways

The Toll, IMD, and JAK-STAT pathways play a central role in controlling the immune response of insects, so we investigated whether they were activated by *B*. *malayi*, and whether patterns of activation differ between resistant and susceptible mosquitoes. To do this we compared our results to microarray datasets from mosquitoes where REL1 or REL2 had been overexpressed (the transcription factors activated by the Toll and IMD pathways respectively) [43] or PIAS, a negative regulator of the JAK-STAT pathway, had been knocked down [44].

**Fig 5:**
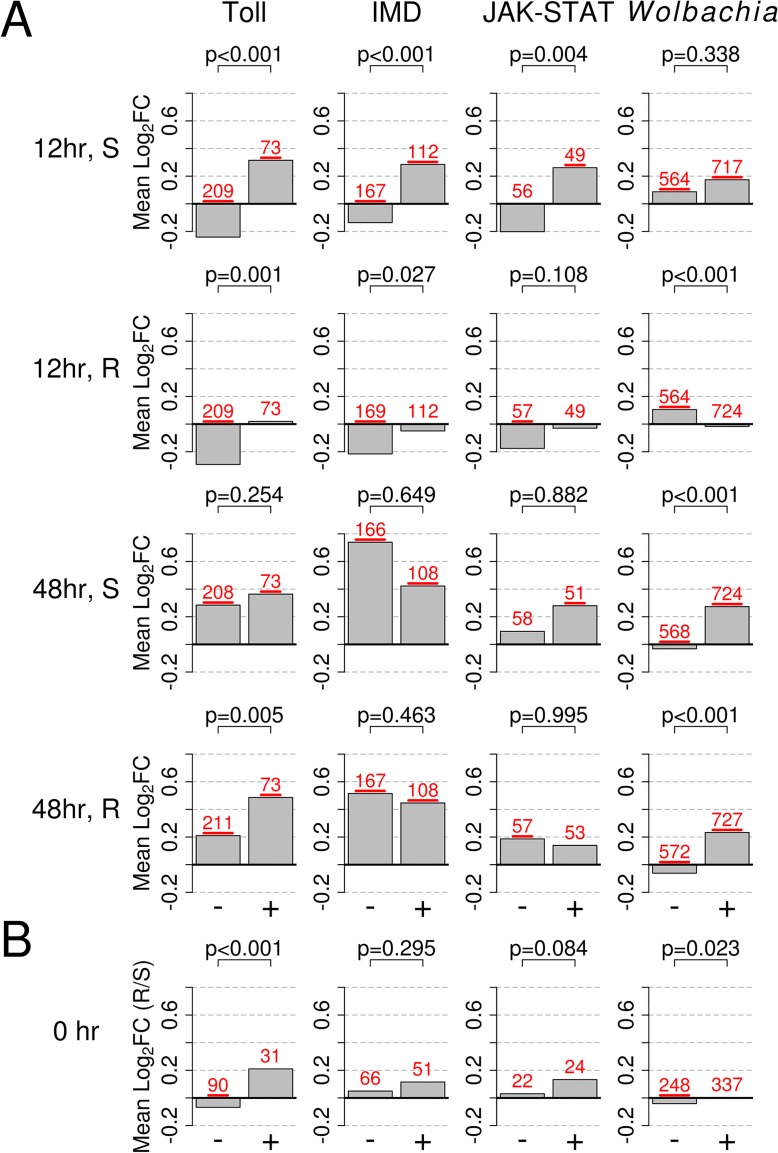
Response to infection of genes in the Toll, IMD, or JAK-STAT pathways, or those responsive to infection with *Wolbachia* strain wMelPop-CLA. The bars show mean log_2_ fold change in expression (Mean Log_2_FC). + or - indicates genes that are upregulated or downregulated by the category shown at the top of each column and were obtained from [43–45]. A) Expression changes in response to *B*. *malayi* in susceptible and resistant genotypes at 12 and 48 hours after infection. B) Constitutive expression of pathways prior to infection (0 hr; resistant versus susceptible), with positive values indicating higher expression in the resistant genotype and vice versa. In B), only genes mapping to the second and third chromosomes, which are not biased with regards to sequence mapping, are shown. The numbers of genes in each category are shown is red above each bar and are underlined if their mean is significantly different from 0 (*t*-test, *P*<0.05). *P*-values from a Mann-Whitney test comparing up and down regulated genes are shown above each bar plot.

At 12 hours post-infection, the transcriptional response of both resistant and susceptible genotypes suggests they have activated their Toll and/or IMD pathways (Figs. 5A, S5). Consistent with our earlier findings of an induced immune response, the susceptible genotype appears to be activating these pathways to a higher degree (Figs. 5A, S5). As has been previously reported, overexpression of REL1 and REL2 elicits similar responses in an overlapping set of genes (S6 Fig) [43], so these patterns could be generated by *B*. *malayi* activating the Toll, IMD or both pathways.

The susceptible genotype appears to activate the JAK-STAT pathway at 12 hours post infection, while the resistant genotype does not show evidence for this (Figs. 5A, S5). In particular, the insect major allergen/G12 gene AAEL009166 appears to be induced in the susceptible line but strongly downregulated by the resistant line. This gene is induced by the JAK-STAT pathway, and is highly expressed in the midgut following a bloodmeal and is thought to be involved in digestion [58,59]. This JAK-STAT-like response is distinct from the other pathways we investigated, as of the 10 genes that overlap between the Toll and JAK-STAT datasets, 9 go in opposite directions (S6 Fig). These 9 genes include genes that respond differently to *B*. *malayi* in the two genotypes: the antimicrobial peptides *CECE* and *DEFC* (upregulated by the Toll pathway and downregulated by the JAK-STAT pathway [44]) and four insect major allergen genes (AAEL009166, AAEL010429, AAEL010436, and AAEL013118; downregulated by the Toll pathway and upregulated by the JAK-STAT pathway).

The pattern of immune pathway activation 48 hours post-infection is less clear. In both the resistant and susceptible mosquitoes, *B*. *malayi* infection increases the expression of genes normally upregulated by active Toll, IMD and JAK-STAT pathways (Figs. 5A, S5). This is reflected in our earlier result that both genotypes show signs of an induced immune response. However, many genes that are normally downregulated by these pathways are upregulated in both genotypes. This upregulation appears to be higher in the susceptible genotype, especially in the case of the IMD pathway (Figs. 5A, S5), including trypsins, a trypsin 5G1 precursor, and serine-type endopeptidases (Fig. 3).

### There Are Strong Correlations between the Transcriptional Responses to *B*. *malayi* and the Bacterial Endosymbiont *Wolbachia*

*Ae*. *aegypti* has been artificially infected with a bacterial symbiont called *Wolbachia* [45], and the wMelPop-CLA strain increases resistance to *B*. *malayi* [21]. Additionally, *B*. *malayi* harbors its own obligate strain of *Wolbachia* wBm [60], which may also interact with the *Ae*. *aegypti* immune response. We therefore compared the transcriptional responses to *Wolbachia* and *B*. *malayi* using previously published data for *Wolbachia* (see Methods).

At the early time point there are striking differences between the resistant and susceptible genotypes. In the resistant genotype there is a strong negative correlation in the responses to *B*. *malayi* and *Wolbachia* wMelPop-CLA, with genes tending to respond in opposite directions (Figs. 5A, S5). In contrast, in the susceptible genotype there is a positive correlation (Figs. 5A, S5).

At 48 hours post-infection, the transcriptional response to *B*. *malayi* in both the susceptible and resistant mosquitoes is strongly correlated to the response to the wMelPop-CLA (Figs. 5A, S5). A subset of these genes behaved differently in the two genotypes, including the downregulation of antimicrobial peptides in susceptible mosquitoes that is described above (S5 Fig).

### Constitutive Differences in Gene Expression between Resistant and Susceptible Genotypes of *Ae*. *aegypti*

Until now we have only investigated induced responses to *B*. *malayi*, but it is possible that constitutive gene expression differences might contribute to resistance. As in the previous section, we identified genes that were upregulated or downregulated by stimulation of the Toll, IMD, or JAK-STAT pathways, or by *Wolbachia* infection (Fig. 5B). We found that the resistant line had significantly higher constitutive expression of genes under the control of the Toll pathway and genes induced by the *Wolbachia* strain wMelPop-CLA (Fig. 5B). Overall, it appears that some immune pathways are upregulated in the resistant genotype prior to infection, suggesting that constitutive differences in gene expression could potentially play a role in resistance.

We confirmed that these results are robust to the confounding effects of biases in mapping sequence reads. Reads will not map to the reference genome if SNPs cause a significant number of mismatches to the reference genome. Despite homogenizing the genetic background of our mosquitoes, the resistant and susceptible genotypes are still genetically different in sex-linked regions. We found that these differences are biasing our estimates of gene expression, as genetic differentiation (*F*_ST_) between the genotypes correlated with differences in expression levels (details in S7 Fig). We therefore repeated our comparison of constitutive expression using only genes found on the second or third chromosomes which are not affected by this bias, and found that the results of our pathway comparisons were qualitatively unchanged. It is important to note that this bias does not affect the analysis of induced responses to *B*. *malayi* as these rely on comparisons within genotype rather than between genotypes.

### Expression Profiles of Candidate Resistance Genes from the Association Study

**Fig 6:**
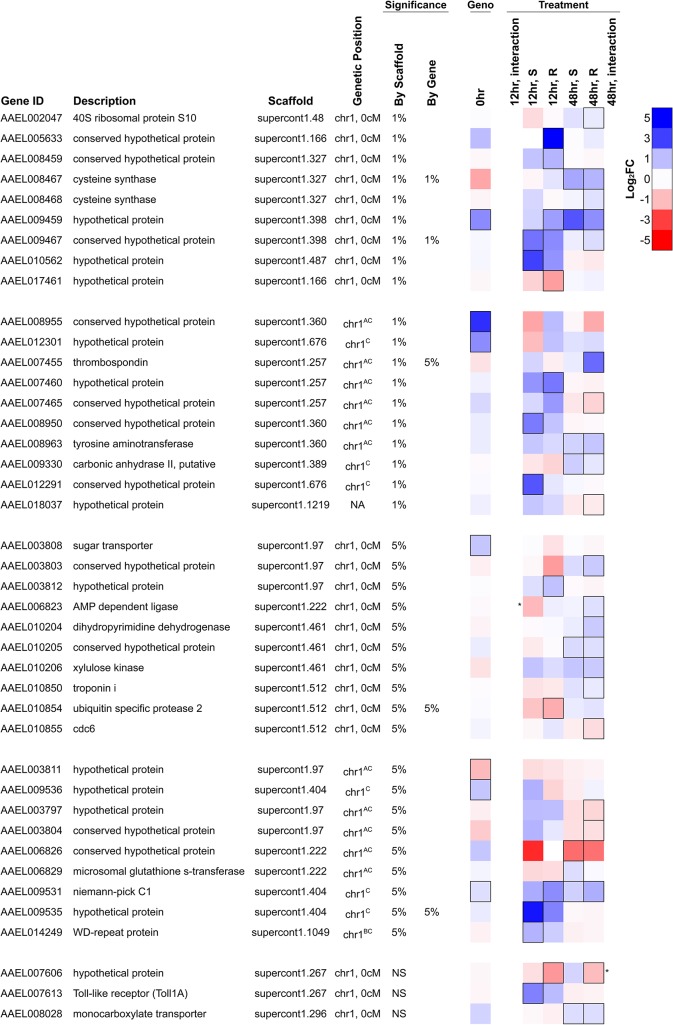
Differentially expressed genes in the genomic region controlling resistance. Genes shown either map to the genetic map position associated with resistance (chromosome 1, 0 cM) or are on an unassembled scaffold with a significant association at a genome-wide significance of *P*<0.01. They are significantly differentially expressed either 1) between resistant and susceptible genotypes prior to infection (FDR<0.20; black border) or 2) in response to infection in either genotype at 12 or 48 hours after infection (FDR<0.20; black border), or 3) respond to infection differently between the genotypes (* indicates a significant interaction between genotype and infection at *P*<0.01). Several genes have been assigned to chromosome 1 but have an unknown genetic map position. ^A^On a misassembled scaffold, ^B^Mapped to chromosome 1 previously without positional information [19], ^C^Newly mapped to chromosome 1 using *F*_ST_ estimates from RNA-seq data. NA indicates no genetic mapping information.

The polymorphism that confers resistance could be in a gene whose expression changes in response to *B*. *malayi* infection. Therefore, we examined our gene expression data for differentially expressed genes in the genomic region controlling resistance (Fig. 6). Specifically, we chose genes that were either differentially expressed in response to *B*. *malayi* infection or constitutively differentially expressed between the genotypes (note the caveat above that this comparison can be confounded by mapping biases). This generated a list of 41 genes (Fig. 6).

The most significant SNP in the association study is within the intron of the gene AAEL009467, and this gene is induced in response to *B*. *malayi* infection by both genotypes at the early time point (non-significant in the resistant genotype) and by the resistant genotype only at the later time point (Fig. 6). This gene encodes a protein that contains an actin-depolymerizing factor homology (ADF-H) domain, which mediates actin binding. It has 1:1 orthologs in other mosquito vectors including *Anopheles gambiae* and *Culex quinquefasciatus*. This SNP is unlikely to be causing resistance as it does not have the expected segregation pattern (see above). We had high sequence coverage and thus high power for this gene across the entire coding sequence so it is unlikely that the casual mutation is protein coding. It is possible that a non-coding variant affecting gene expression could be causal.

An unnamed gene (AAEL009459) encoding a hypothetical protein is located 80kb away from the most significant hit and is strongly induced by infection in resistant mosquitoes at the early time point and in both genotypes at the later time point (Fig. 6). It also has higher constitutive expression in the resistant genotype, although high *F*_ST_ suggests that this may be an artifact of mapping biases. This gene is also strongly upregulated by *Wolbachia* wMelPop-CLA and has no known orthologs in other species. This gene appears to have a neighboring unannotated paralog that is expressed in our RNA-seq data, and the duplicate has two distinct alleles in the resistant and susceptible genotypes from the laboratory crosses. Unfortunately, because it is unannotated, sequence coverage is extremely low in the association study samples, so it is unclear if these alleles are associated with resistance.

Two immune-related genes, a Niemann Pick-type C1 (*NPC1*) gene (AAEL009531) and *Toll1A* (AAEL007613), are also found in the region associated with resistance and were upregulated in response to infection in one or both genotypes. The Niemann Pick-type C1 gene is expressed in the mosquito midgut [61] and here is induced in response to *B*. *malayi* infection. Knockdown of this gene inhibits dengue infection, and it has been proposed to be a negative regulator of the immune response [61]. *Toll1A* is a homolog to the Drosophila *Toll* [62], and the scaffold 1.267, which contains *Toll1A*, also contains several other *Toll* genes.

## Discussion

We have found that a single, dominant locus underlies resistance to *B*. *malayi* in a wild outcrossed population of *Ae*. *aegypti* from Kenya. The locus maps to the same region of the genome as the *f* ^*m*^ locus [12,18], which was identified in laboratory lines established from West Africa in 1936, suggesting that this laboratory-observed variant is present at appreciable frequencies in the wild and has been maintained for at least 80 years. By exploiting the power of exome sequencing to allow association studies in large and repetitive genomes, we have narrowed the causal region to a single genetic position and identified candidate resistance genes. By studying gene expression in resistant and susceptible genotypes, we found a reduced immune response in the susceptible compared to the resistant genotype at the time of parasite killing, which could potentially result from the parasite suppressing host immunity. We have also found differences in the expression of reproductive and digestion related genes, suggesting that resource allocation may be affected differently between the genotypes.

### Resistance to *B*. *malayi* Has a Simple Genetic Basis

Resistance of *Ae*. *aegypti* to *B*. *malayi* has a simple genetic basis. By combining exome and whole-genome sequencing, we found that resistance to *B*. *malayi* in a Kenyan population of *Ae*. *aegypti* is controlled by a single dominant locus. This locus is located in the same genomic region as a dominant QTL controlling *B*. *malayi* resistance that was mapped in crosses between laboratory lines from West Africa [12,18]. In quantitative genetics it is commonly seen that major-effect QTL from lab crosses are an artifact caused by multiple closely linked loci of smaller effect [63], and these are broken up using high-resolution approaches like ours. We found that a model accounting for the most significant SNP gave no evidence for any additional associations, which is most parsimoniously explained by resistance to *B*. *malayi* being controlled by a single causative locus. An alternative explanation is that multiple resistance genes are being held in tight linkage disequilibrium by an unknown chromosomal inversion. It is also normal in QTL studies to identify different loci when using different crosses [64], and for QTL that are important in the laboratory to be unimportant in the wild [65,66]. Again that appears not to be the case here, as we found that the locus identified from West Africa is common in an East African population.

The simple genetics of *B*. *malayi* resistance is striking as most quantitative traits tend to be affected by many genes, each of which only has a small phenotypic effect [63]. However, there are a number of studies suggesting that susceptibility to infection in insects often has a very simple genetic basis. For example, in *Drosophila* a small number of major effect loci control resistance to viruses [67], and the same is probably true for resistance to parasitoid wasps [68,69]. Similarly, it has been suggested that in humans susceptibility to infection has a simpler underlying genetic basis than other quantitative traits due to strong selection by pathogens [70]. It has been suggested that this simple genetics is a result of directional selection driving major effect resistance alleles through populations [67].

The polymorphism we have investigated in African mosquitoes is not the result of *B*. *malayi* selecting for resistance, because the parasite is only found in south and south-east Asia and is not thought to be naturally vectored by *Ae*. *aegypti*. It is known that this locus also confers resistance to the filarial nematodes *B*. *pahangi* and *Wuchereria bancrofti*, but not to *Dirofilaria immitis*, the cause of dog heartworm [16]. It is possible that selection for resistance to another closely related but unidentified nematode is responsible for the maintenance of this polymorphism. Any selection for resistance is likely to vary among populations, as genotypes that are susceptible to *B*. *pahangi* are only known to be common in East Africa [15,71]. Thus it remains to be seen what has maintained this polymorphism in the wild since at least 1936, but understanding this experimentally tractable system may yield insights into natural mosquito-parasite associations. In mosquito-filarial systems, it is commonly seen that resistance is sex-linked and dominant, including a separate locus that confers resistance of *Ae*. *aegypti* to *D*. *immitis* and to the bullfrog filarial nematode *Waltonella flexicauda* [72,73]. It is tempting to speculate that this could be the result of sexually-antagonistic selection favoring resistance only in females.

### Towards Identifying the Resistance Gene

Our association study mapped the locus causing resistance with a high precision, but we did not identify the causative gene. Due to large effective population sizes, linkage disequilibrium in insect populations can sometimes extend only a few tens of base pairs [74]. This means that association studies like ours would often be expected to identify the exact genes involved. However, we found high levels of linkage disequilibrium in our mapping population and multiple loci with identical segregation patterns. This could be due to difficulties in establishing African *Ae*. *aegypti* in the laboratory resulting in a relatively small number of individuals founding our mapping population. This could also occur if the resistance locus falls within a chromosomal inversion, which may result in long-distance linkage disequilibrium in natural populations and might prevent mapping to the level of a single gene. Despite this, we localized the locus with a very high resolution to a single genetic position (0 cM) and identified a small number of strong candidate genes, one of which may cause resistance. The availability of detailed genetic maps combined with the density of genetic markers allowed by whole-exome sequencing has enabled us to compile the first complete list of candidate resistance genes. Several steps are needed to identify the exact variant(s) causing resistance. First, additional association studies on populations with less linkage disequilibrium may narrow identify far fewer genes. Second, gene knockouts, RNAi and other functional studies can confirm the role of these genes in defense against filarial worms.

In our association study, we identified polymorphisms in five different genes which best explained resistance and had the segregation pattern expected for single dominant resistance locus. None of these genes have any known involvement in the immune response, but it is still possible that one of them is responsible for resistance. We also identified candidates in the correct region of the genome with expression differences between the resistant and susceptible genotypes or in response to infection. These candidates did not always have significant associations, but it is possible that the true causative site was untested if it is non-coding or lacked sufficient coverage. Two genes identified this way, a niemann-pick C1 gene and *Toll1A*, are thought to be involved in the immune response. The niemann-pick C1 gene, which is upregulated in response to *Brugia* infection, has recently been shown to be required for successful dengue infection [61]. *Toll1A* is one of four *Ae*. *aegypti* genes homologous to *Drosophila Toll* (*Toll-1*) [62,75], and it is induced following infection. In *Drosophila* Toll is crucial for signaling through the Toll pathway, and while Toll1A does not play the same role in *Ae*. *aegypti*, there is some evidence to suggest it may play a role in modulating the immune response [62]. Lastly, the top hit in our association study is an unnamed gene that is induced by infection (AAEL009467). This gene encodes a protein with an actin-binding ADF-H domain and *Brugia* develops in the thoracic muscle fibers of *Ae*. *aegypti*, where actin dynamics are expected to be important.

### The *Ae*. *aegypti* Immune Response to *B*. *malayi* Differs in Resistant and Susceptible Mosquitoes

We found that *Ae*. *aegypti* upregulates immune-related genes in response to *B*. *malayi* as early as 12 hours after infection. Many of these immune genes are known to be under the control of the Toll and/or IMD signaling pathways, so it is likely that *B*. *malayi* is activating one or both of these pathways (although the pattern is less clear-cut if genes downregulated by these pathways are considered; see Results). The role of the immune response in resistance to filarial infection is unclear, as it has only been studied in susceptible mosquitoes and the outcome of those studies is not entirely clear-cut. Activation of the immune system prior to infection with *B*. *malayi* confers a protective effect, with susceptible *Ae*. *aegypti* mosquitoes that are given sham infections or non-lethal bacterial infections sustaining a significantly lower intensity of infection [20]. Similarly, infection with the bacterium *Wolbachia* wMelPop-CLA also induces an immune response and provides some protection to susceptible mosquitoes [21]. However, RNAi knockdown of the Toll pathway and over-activation of the Toll or IMD pathways in susceptible mosquitoes did not influence the response to infection [11]. This could simply be that using RNAi to induce an immune response is less efficient than using bacteria, or it may mean Toll- and IMD-independent aspects of immunity are controlling filarial infections. Only by repeating these assays in resistant mosquitoes will it be possible to determine which pathways underlie protection in genetically resistant individuals. At 48 hours after infection, the genes most responsive to *B*. *malayi* infection are those that are induced by *Wolbachia* wMelPop-CLA infection. It is possible that the mosquito immune response may be launched not solely against the worm itself, but also in response to the *Wolbachia* harbored by the nematode, as is the case in the vertebrate host [76], or to midgut bacteria that enter the hemocoel after *B*. *malayi* penetration.

By comparing gene expression in both resistant and susceptible mosquitoes, we find that resistant mosquitoes are upregulating a number of immune effector genes at the time point where the parasites are dying, whereas the susceptible mosquitoes are not. At 12 hours post infection the immune response of the two genotypes is similar, but by 48 hours post infection, when parasites are dying in resistant mosquitoes, differences in the immune response are clear. At 48 hours, effector molecules of the immune response, including anti-microbial peptides (AMPs) and prophenoloxidases (PPOs), are upregulated in resistant mosquitoes and either downregulated in susceptible mosquitoes or induced to a much lower degree. Expression of the AMP cecropin was previously seen to be downregulated 48–72 hours after infection in susceptible mosquitoes, supporting what we’ve seen here [11]. Although AMPs are often thought to be primarily antibacterial and antifungal, cecropins have been shown to kill *B*. *panhangi* in vitro and in vivo [22], and cecropin and defensin overexpression kills *Plasmodium* in *Ae*. *aegypti* [77].

The downregulation of immune effectors like antimicrobial peptides in susceptible mosquitoes suggests that the differences in the immune response may be driven by immune suppression by the parasite. Parasitic nematodes have mechanisms that circumvent the humoral immune responses of their hosts, including proteinases that directly inhibit cecropins and cuticle factors that sequester host hemolymph proteins and prevent the activation of the immune response (reviewed in [78]). This is likely to be the case for *B*. *malayi*, as the closely related parasite *B*. *pahangi* reduces the melanization response of *Ae*. *aegypti* [79]. If parasite suppression of the immune response is causing the weaker immune response seen in susceptible mosquitoes, then this pattern could either be a cause or consequence of resistance. The resistance locus that we mapped might cause the mosquitoes to mount a stronger immune response to *B*. *malayi*, perhaps by circumventing the parasite suppression, and this in turn is the cause of resistance. Alternatively nematodes in the resistant mosquitoes may be unable to suppress the immune response because they are dying for some other reason, such as if resistant mosquitoes are metabolically inhospitable or lack a receptor necessary for nematode migration. In this case, the stronger reduction in immune response seen in susceptible mosquitoes may be a consequence of having more and healthier nematodes.

Resistant mosquitoes also constitutively express immune genes to a higher level, which supports a direct role for the immune system in resistance. We found that resistant mosquitoes have higher constitutive expression of genes under the control of the Toll pathway as well as genes expressed in response to infection with *Wolbachia* wMelPop-CLA. As described earlier, both experimental stimulation of the immune response and infection with *Wolbachia* strain wMelPop-CLA provide some protection to *Brugia* infection, although not complete resistance [20,21].

### *B*. *malayi* Alters the Expression of Genes Involved in Digestion, Nutrient Transport and Egg Production

Genes involved in digestion, nutrient transport and egg production are expressed differently between the resistant and susceptible mosquitoes after infection by *B*. *malayi*. Susceptible mosquitoes appear to be upregulating genes involved in digestion and nutrient transport, and downregulating the egg yolk precursor protein vitellogenin. Many of the genes that are differently induced are genes that respond to blood feeding [58]. The target of rapamycin (TOR) pathway is central to coordinating this blood-feeding response, and is thought to detect the influx of amino acids and upregulate the expression of genes related to digestion, nutrient transport, and vitellogenesis [80]. Several of the genes that respond differently to infection in the two genotypes are in or under the control of the TOR pathway, including a known translational repressor of the pathway 4E-BP [55], the amino acid transporter PAT1 [81], and Vitellogenin A1 precursors [80]. Amino acids and other nutrients that are ingested in a blood meal are normally used for egg production. In infected mosquitoes, competition between the host and parasite for nutrients can result in a reduction of egg production [82], such as seen with *Wolbachia* [83] and *Plasmodium* [84]. There can also be a conflict between the immune system and egg production, where the same resources are needed for both processes [85]. Altered expression of these genes could reflect active or passive manipulation of the host resources by the parasite, or competition within the mosquito for resources.

Vitellogenin expression is strongly downregulated by *B*. *malayi* in susceptible mosquitoes at 12 hours post-infection and strongly upregulated in resistant mosquitoes at 48 hours-post infection. Despite this, no difference in the number of eggs laid is seen in either genotype in response to infection (Cristina Ariani, personal communication), although other mosquito-filarial worm combinations do lead to a reduction [85–87]. In *An*. *gambiae* mosquitoes, vitellogenin production is negatively regulated downstream of Cactus/REL1/REL2, leading to the arrest of oogenesis when the immune system in activated [88]. Vitellogenin interferes with TEP1 binding to the surface of developing *Plasmodium* ookinetes, so downregulation of vitellogenin production boosted the efficiency of parasite killing [88].

A key molecule underlying the different responses to *B*. *malayi* in resistant and susceptible mosquitoes might be trypsin modulation oostatic factor (TMOF). TMOF is produced in the ovaries and turns off egg production and late trypsin production by binding to midgut receptors [56]. Susceptible mosquitoes have decreased TMOF production at 12 hours, and are expressing trypsins and serine proteases at higher levels at 48 hours. Trypsin production in the midgut is highly upregulated by blood feeding to allow quick digestion of proteins in the blood meal [57], and tryptic activity has long been speculated to be a source of conflict with the parasite. Trypsin activity has been suggested to change nutrient availability, or to either digest or activate parasite proteins as they pass through the midgut [89,90]. Indeed, inhibition of trypsin in *Ae*. *aegypti* increases dengue virus titres [89] and decreases *Plasmodium* load [91].

In conclusion, by developing exome sequencing we were able to conduct the first large-scale association study in *Ae*. *aegypti*, a species with a large and repetitive genome. This allowed us to identify a major-effect locus conferring resistance to *B*. *malayi*. This locus alters the transcriptional response of mosquitoes to *B*. *malayi*, with resistant individuals expressing immune effectors to a higher degree. Our results suggest that this pattern may be driven by *B*. *malayi* suppressing the immune response in susceptible mosquitoes.

## Supporting Information

S1 Fig Quantile-quantile (qq) plots of *P*-values from the *B*. *malayi-*resistance association study. The observed *P*-values are plotted against the *P*-values expected if there are no true associations. Black dots show observed *P*-values from the association study based on the additive model (see main text). Red dots show the observed *P*-values when the most significant SNP was included as a covariate. The dotted line shows the relationship that is expected in the absence of an association.
(TIF)

S2 Fig Genetic differentiation between the resistant and susceptible genotypes used to measure gene expression. We used a backcross which resulted in the first chromosome of *Ae*. *aegypti* being differentiated between resistant and susceptible genotypes, while the second and third chromosomes were not. The differentiation extends across the length of the first chromosome. Mean *F*_ST_ estimated from RNA-seq data is shown for each centimorgan (cM) position, and the number of genes measured at each position is shown in red. Scaffolds that contained one or more gene with *F*_ST_ greater than 0.1 and with at least 10 segregating sites were considered to be misassembled and moved to the unassembled region for all analyses.
(TIF)

S3 Fig Genes from selected functional categories that respond differently to *B*. *malayi* infection in resistant and susceptible mosquitoes. Heatmaps represent the log_2_ fold changes in response to infection. Black boxes indicate that the gene is differentially expressed in response to *B*. *malayi* with an FDR<0.2. Asterisks (*) indicate a genotype by infection-status interaction at *P*<0.01, where susceptible and resistant mosquitoes respond to infection with expression changes of significantly different magnitudes or directions.
(PDF)

S4 Fig Gene ontology terms enriched among genes that are differentially expressed in response to *B*. *malayi* infection. Statistical significance is represented in green, and the mean log_2_ fold change in blue (up in infected) or red (down in infected). R: resistant genotype; S: susceptible genotype; I: difference between the genotypes in response to *B*. *malayi* (the interaction of genotype and infection). Only ontologies involved in biological process are shown.
(PDF)

S5 Fig Correlation between changes in gene expression in response to *B*. *malayi* infection and the response to Toll or IMD pathway activation or infection with the bacterial symbiont *Wolbachia* wMelPop-CLA. Expression is given as log_2_ fold change in response to infection or pathway activation. Blue and red points behave significantly different in the two genotypes in response to infection at *P*<0.01 at 12 hr (blue square) or 48 hr (red triangle) after infection. Dotted lines indicate best fit lines for all data points. Genes believed to be involved in the immune response (those in the immunodb database or manually curated) are labeled. A subset of genes induced by infection by the *Wolbachia* strain wMelPop-CLA are induced in susceptible mosquitoes but downregulated by resistant mosquitoes at 12 hours after infection. Genes in this category include CECN (AAEL000621), 4E-BP (AAEL001864), cAMP-dependent protein kinase catalytic subunit (AAEL007293), endothelin-converting enzyme (AAEL011369), and integrin beta subunit (AAEL012466).
(TIF)

S6 Fig Comparison between the genes that are differentially expressed by activation of the Toll, IMD, JAK-STAT pathways or infection by *Wolbachia*. Toll and IMD pathway activities were determined by overexpression of the transcription factors REL1 and REL2 respectively, and JAK-STAT activity was determined by knockdown of the negative regulator PIAS. The number of genes in each category are shown is red above each bar. + or - indicates genes that are upregulated or downregulated by the category shown at the top of each column and were obtained from [43–45]. Statistical significance is from a Mann-Whitney test.
(TIF)

S7 Fig Estimates of constitutive gene expression differences between resistant and susceptible genotypes are confounded by mapping biases. Log_2_FC is the comparison of resistant vs susceptible gene expression, and is greater than 0 if resistant mosquitoes have higher expression. The left panel shows the correlation between genetic differentiation (*F*_ST_) and differential expression. The right panel shows the mean differential expression at different levels of *F*_ST_ (number of genes in red). In genes that are highly genetically differentiated (high *F*_ST_) between the resistant and susceptible genotypes there tends to be higher constitutive expression in the resistant genotype. We mapped sequence reads to the published genome sequence, which is from the resistant LVP^R^ line we used to generate these mosquitoes. This indicates that a false signature of constitutive expression differences results from a bias in being more likely to map reads that match the published genome.
(TIF)

S1 Table Genome and exome sequencing design. (PDF)

S2 Table RNA sequencing design. Libraries were named by time point, mosquito genotype, infection status (if applicable), and cage number. The # of reads obtained refers to the numbers of reads post-quality trimming.
(PDF)

S3 Table Biological coefficients of variation (BCV) for RNA-seq experiment. (PDF)

S1 Dataset Association mapping results. (XLSX)

S2 Dataset RNA-seq results. Differential expression of genes in *Aedes aegypti* in response to infection by *Brugia malayi* at time points shortly after infection; genes with a significant interaction between genotype and infection status at time points shortly after infection; and differential expression of genes between resistant and susceptible genotypes of *Ae*. *aegypti* prior to infection.
(XLSX)
