# Supplementary figures and images for: Exome and Transcriptome Sequencing of Aedes aegypti Identifies a Locus That Confers Resistance to Brugia malayi and Alters the Immune Response
Source: PLoS Pathog. 2015 Mar 27;11(3):e1004765. doi: 10.1371/journal.ppat.1004765 (PMC4376896; doi:10.1371/journal.ppat.1004765)

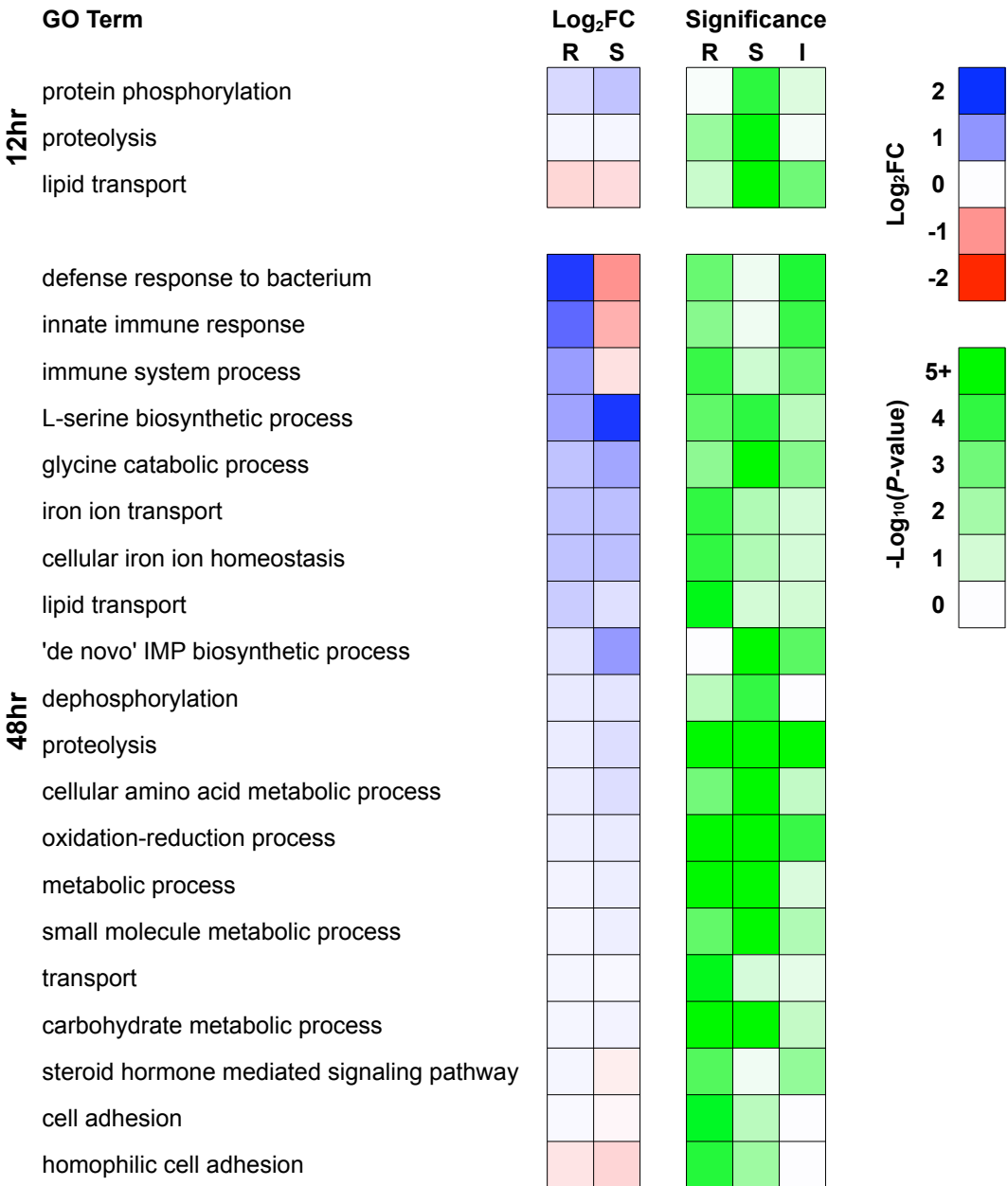

Supplement: S4 Fig — Statistical significance is represented in green, and the mean log2 fold change in blue (up in infected) or red (down in infected). R: resistant genotype; S: susceptible genotype; I: difference between the genotypes in response to B. malayi (the interaction of genotype and infection). Only ontologies involved in biological process are shown. (PDF) [file ppat.1004765.s004.pdf]
