# Supplementary material for: Exome and Transcriptome Sequencing of Aedes aegypti Identifies a Locus That Confers Resistance to Brugia malayi and Alters the Immune Response
Source: PLoS Pathog. 2015 Mar 27;11(3):e1004765. doi: 10.1371/journal.ppat.1004765 (PMC4376896; doi:10.1371/journal.ppat.1004765)
Supplement: S1 Table — (PDF) [file ppat.1004765.s008.pdf]

Table S1. Genome and exome sequencing design.

Lane #	Individuals	Library Type	Target	Sequencing
1	*169R 171S 172S 174S*<br>*180R 181R 186R 187R*<br>*189S 190S 194S 195R*<br>*199R 201R 210R 211R*<br>*215S 220S 221R 222S*<br>*226S*<br>(n=21)	Nextera	Whole genome	100bp PE HiSeq<br>(KAUST)
2	*169R 171S 172S 174S*<br>*180R 181R 186R 187R*<br>*189S 190S 194S 195R*<br>*199R 201R 210R 211R*<br>*215S 220S 221R 222S*<br>*226S*<br>(n=21)	Nextera	Whole genome	100bp PE HiSeq<br>(KAUST)
3	21R 22R 24S 27R 30S 35R<br>44S 45R 49S 52R 60R 61S<br>67S 71R 90S 91S 124S<br>269S 350R *370R* 374R<br>385S 386R 408R<br>(n=24)	Nextera	Whole genome	100bp PE HiSeq<br>(KAUST)
4	20S 23S 31R 36S 66S 74R<br>76R 77S 87R 89R 119S<br>127R 147S 152R 198R<br>219R 225S 227R 278R<br>348S 359R 361R 362S<br>*381S* 400S<br>(n=25)	Nextera	Whole genome	100bp PE HiSeq<br>(KAUST)
5	93S 97S 99S 102R 103R<br>104R 105R 112R *370R*<br>371S 372S 377S 379R<br>380R *381S* 382S 383R<br>387R 398R 399S 405S<br>406R 409R 411S<br>(n=24)	TruSeq	Exome	150bp PE MiSeq<br>(Oxford)
6	94S 96R 98S 100R 106R<br>213R 216S 218S 233R<br>235S 236S 240R 256R<br>259R 260S 261S 301R<br>313S 315R 316S 324S<br>326R 384S 391R<br>(n=24)	TruSeq	Exome	100bp PE HiSeq<br>(Oxford)
7	101S 107S 108S 110S<br>113R 114R 166S 167R<br>196R 197S 200S 212R<br>271S 281S 283R 288R<br>292S 298R 299R 303R<br>307R 308S 311S 314R<br>(n=24)	TruSeq	Exome	100bp PE HiSeq<br>(Oxford)

Individuals in italics were sequenced twice.
