# Supplementary material for: Exome and Transcriptome Sequencing of Aedes aegypti Identifies a Locus That Confers Resistance to Brugia malayi and Alters the Immune Response
Source: PLoS Pathog. 2015 Mar 27;11(3):e1004765. doi: 10.1371/journal.ppat.1004765 (PMC4376896; doi:10.1371/journal.ppat.1004765)
Supplement: S2 Table — Libraries were named by time point, mosquito genotype, infection status (if applicable), and cage number. The # of reads obtained refers to the numbers of reads post-quality trimming. (PDF) [file ppat.1004765.s009.pdf]

Table S2. RNA sequencing design. Libraries were named by time point, mosquito genotype, infection status (if applicable), and cage number. The # of reads obtained refers to the numbers of reads post-quality trimming.

Lane #	Libraries	# of Reads Obtained
1	48hr-S-Brugia-C1	36,180,742
	48hr-R-Brugia-C2	32,883,742
	48hr-S-Control-C3	33,478,626
	48hr-R-Control-C4	42,715,370
	48hr-S-Brugia-C5	51,356,958
	48hr-R-Brugia-C6	36,339,868
	48hr-S-Control-C7	58,400,516
	48hr-R-Control-C8	41,325,686
	0hr-S-C5	48,537,302
	0hr-R-C6	34,887,180
2	48hr-S-Brugia-C9	41,106,956
	48hr-R-Brugia-C10	32,388,940
	48hr-S-Control-C11	58,865,542
	48hr-R-Control-C12	31,697,882
	48hr-S-Brugia-C13	37,819,770
	48hr-R-Brugia-C14	40,384,214
	48hr-S-Control-C15	42,983,224
	48hr-R-Control-C16	41,957,676
	0hr-S-C7	29,884,246
3	12hr-S-Brugia-C1	56,807,888
	12hr-R-Brugia-C2*	36,637,808
	12hr-S-Control-C3	36,380,274
	12hr-R-Control-C4	45,763,976
	12hr-S-Brugia-C5	33,957,732
	12hr-R-Brugia-C6	38,347,362
	12hr-S-Control-C7	36,144,138
	0hr-S-C1	45,432,462
	0hr-R-C2	76,500,644
4	12hr-S-Brugia-C9*	23,721,280
	12hr-R-Brugia-C10*	28,026,154
	12hr-S-Control-C11	49,146,766
	12hr-R-Control-C12	43,920,258
	12hr-S-Brugia-C13*	40,488,014
	12hr-R-Brugia-C14	40,028,056
	12hr-R-Control-C16	47,825,728
	0hr-R-C4	70,624,632

*These libraries were excluded from the final analysis.
