# Supplementary material for: Exome and Transcriptome Sequencing of Aedes aegypti Identifies a Locus That Confers Resistance to Brugia malayi and Alters the Immune Response
Source: PLoS Pathog. 2015 Mar 27;11(3):e1004765. doi: 10.1371/journal.ppat.1004765 (PMC4376896; doi:10.1371/journal.ppat.1004765)
Supplement: S3 Table — (PDF) [file ppat.1004765.s010.pdf]

Table S3. Biological coefficients of variation (BCV) for RNA-seq experiment.

	BCV	# of Genes
**1) Treatment**		
12hr, S	0.455/0.353	12,255/12,559
12hr, R	0.409/0.301	12,292/12,549
48hr, S	0.230	12,944
48hr, R	0.179	13,137
**2) Constitutive**		
0hr	0.187	13,121
**3) Interaction**		
12hr	0.3058/0.232	12,945/13,156
48hr	0.161	13,594

12hr values are shown before/after removal of low quality RNA-seq libraries.
