# Supplementary material for: Exome and Transcriptome Sequencing of Aedes aegypti Identifies a Locus That Confers Resistance to Brugia malayi and Alters the Immune Response
Source: PLoS Pathog. 2015 Mar 27;11(3):e1004765. doi: 10.1371/journal.ppat.1004765 (PMC4376896; doi:10.1371/journal.ppat.1004765)
Supplement: S3 Fig — Heatmaps represent the log2 fold changes in response to infection. Black boxes indicate that the gene is differentially expressed in response to B. malayi with an FDR<0.2. Asterisks (*) indicate a genotype by infection-status interaction at P<0.01, where susceptible and resistant mosquitoes respond to infection with expression changes of significantly different magnitudes or directions. (PDF) [file ppat.1004765.s003.pdf]

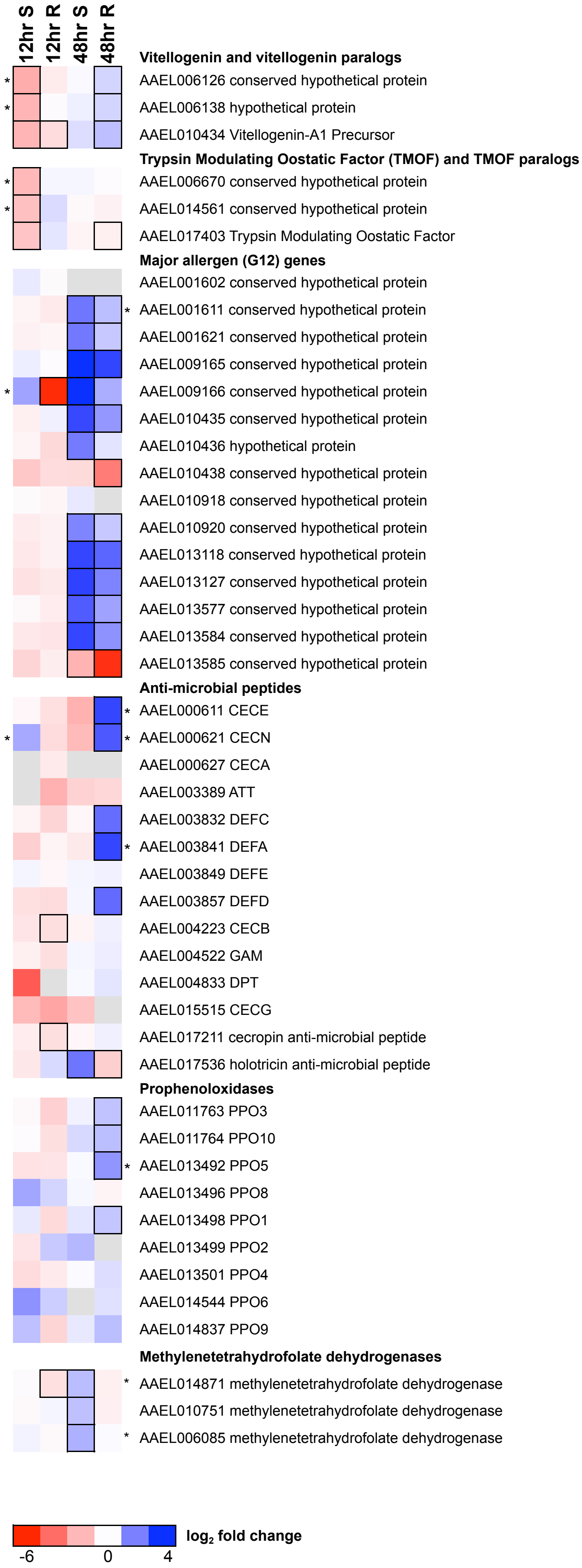
12hr S
12hr R
48hr S
48hr R
Vitellogenin and vitellogenin paralogs
AAEL006126 conserved hypothetical protein
AAEL006138 hypothetical protein
AAEL010434 Vitellogenin-A1 Precursor
Trypsin Modulating Oostatic Factor (TMOF) and TMOF paralogs
AAEL006670 conserved hypothetical protein
AAEL014561 conserved hypothetical protein
AAEL017403 Trypsin Modulating Oostatic Factor
Major allergen (G12) genes
AAEL001602 conserved hypothetical protein
AAEL001611 conserved hypothetical protein
AAEL001621 conserved hypothetical protein
AAEL009165 conserved hypothetical protein
AAEL009166 conserved hypothetical protein
AAEL010435 conserved hypothetical protein
AAEL010436 hypothetical protein
AAEL010438 conserved hypothetical protein
AAEL010918 conserved hypothetical protein
AAEL010920 conserved hypothetical protein
AAEL013118 conserved hypothetical protein
AAEL013127 conserved hypothetical protein
AAEL013577 conserved hypothetical protein
AAEL013584 conserved hypothetical protein
AAEL013585 conserved hypothetical protein
Anti-microbial peptides
AAEL000611 CECE
AAEL000621 CECN
AAEL000627 CECA
AAEL003389 ATT
AAEL003832 DEFC
AAEL003841 DEFA
AAEL003849 DEFE
AAEL003857 DEFD
AAEL004223 CECB
AAEL004522 GAM
AAEL004833 DPT
AAEL015515 CECG
AAEL017211 cecropin anti-microbial peptide
AAEL017536 holotricin anti-microbial peptide
Prophenoloxidases
AAEL011763 PPO3
AAEL011764 PPO10
AAEL013492 PPO5
AAEL013496 PPO8
AAEL013498 PPO1
AAEL013499 PPO2
AAEL013501 PPO4
AAEL014544 PPO6
AAEL014837 PPO9
Methylenetetrahydrofolate dehydrogenases
AAEL014871 methylenetetrahydrofolate dehydrogenase
AAEL010751 methylenetetrahydrofolate dehydrogenase
AAEL006085 methylenetetrahydrofolate dehydrogenase
log2 fold change
-6
0
4
